# Low-dose trimethoprim-sulfamethoxazole for prophylaxis of *Pneumocystis jirovecii* pneumonia in HIV-uninfected patients: a systematic review and meta-analysis

**DOI:** 10.3389/fphar.2025.1545436

**Published:** 2025-07-15

**Authors:** Hui-Bin Huang, Jia-Heng Shi, Yan-Ge Hu, Yi-Bing Zhu, Da-Xing Yu

**Affiliations:** Department of Critical Care Medicine, Guang’anmen Hospital, China Academy of Chinese Medical Sciences, Beijing, China

**Keywords:** *Pneumocystis jirovecii* pneumonia, trimethoprim-sulfamethoxazole, discontinuation rate, prophylaxis, meta-analysis

## Abstract

**Background:**

Trimethoprim-sulfamethoxazole (TMP-SMX) is the recommended first-line prophylactic agent against *Pneumocystis jirovecii* pneumonia (PJP). However, the standard regimen is often discontinued due to its drug-associated adverse events (AEs), especially in immunocompromised patients without HIV infection. Therefore, we aimed to investigate the efficacy and safety of a low-dose regimen of TMP-SMX against PJP prophylaxis in patients without infection.

**Methods:**

We searched PubMed, Embase, Wanfang, China National Knowledge Infrastructure, Web of Science, and the Cochrane database for relevant articles from inception to 15 October 2024. Studies were included if they reported the safety and efficacy of using TMP-SMX in PJP prophylaxis in patients without HIV infection. The primary outcome was the discontinuation rate. We assessed study quality and performed sensitivity and subgroup analysis to explore potential heterogeneity among the included studies.

**Results:**

Seventeen studies with 4,890 patients were included. These studies were low to modest in quality. Overall, the incidence of PJP in the included studies was rare and was similar between the low- and standard-dose groups. However, the low-dose regimen significantly reduced the risk of discontinuation rate (odds ratio [OR] = 0.38; 95% CI, 0.27–0.52; *I* ^2^ = 0%; *P* < 0.00001). Further sensitivity and subgroup analyses confirmed this finding. Estimation of the combined discontinuation rate for patients receiving low-dose TMP-SMX was 10% (95% CI, 4%–16%). The low-dose regimen also significantly reduced total AEs (OR = 0.33; 95% CI, 0.24–0.46; *I* ^2^ = 22%; *P* < 0.00001) and improved the incidence of most specific AEs (ORs ranged from 0.24 to 0.67), especially in outcomes of fever, rash, thrombocytopenia, hyponatremia, and liver and renal function (*P* values ranged from 0.0001 to 0.02).

**Conclusion:**

Our findings suggested that a low-dose TMP-SMX regimen is safe and significantly reduces the discontinuation rate and total AEs compared to the standard regimen against PJP in HIV-uninfected patients. Thus, it is a potentially promising prophylactic regimen, and more well-designed, high-quality research should be conducted.

**Systematic Review Registration:**

https://inplasy.com/inplasy-2024-4-0084/.

## Introduction

*Pneumocystis jiroveci* pneumonia (PJP) is a potentially life-threatening opportunistic infection that occurs in both patients with human immunodeficiency virus (HIV)-infected and immunocompromised patients without HIV infection (Kuruvilla and de la Morena, 2013; Katsuyama et al., 2014). The latter increases in solid organ transplant recipients, rheumatic diseases, long-term hormone therapy, and biological immunotherapy. In their study, Monnet and colleagues reported that among all patients with PCP to the ICU, the proportion of HIV-negative cases increased from 0% in 1993 to 75% in 2006 (Monnet et al., 2008). Once these patients are infected with PJP, their mortality rate (48%–52.9%) is much higher than in patients with HIV infection (0%–17%) (Monnet et al., 2008; Enomoto et al., 2010; Ward and Donald, 1999). Therefore, it is very essential for PJP prophylaxis in HIV-uninfected patients. Several drugs are available for PJP prophylaxis, with trimethoprim-sulfamethoxazole (TMP-SMX) being the recommended first-line prophylactic regimen for PJP (Martin et al., 2013; Taplitz et al., 2018; Tomblyn et al., 2009).

The standard dose of TMP-SMX for PJP prophylaxis consists of one single-strength (SS) tablet (80 mg/400 mg) per day or three double-strength (160 mg/800 mg) tablets per week, that is, 6–14 SS tablets per week are considered the standard dose (Martin et al., 2013; Taplitz et al., 2018; Tomblyn et al., 2009). The PJP prophylaxis is usually taken for a long time or even a lifetime, depending on the patient’s disease condition (Ghembaza et al., 2020; Kim et al., 2019). Research has shown that TMP-SMX has a high rate of PJP prevention in patients without HIV infection and significantly reduced PJP-associated mortality (Green et al., 2007). However, TMP-SMX prophylaxis can often cause high risks of adverse events (AEs), as shown in previous studies (39.2%–58.6%) in this patient population (Pope et al., 2003; Utsunomiya et al., 2017). The AEs included fever, rash, electrolyte abnormalities, renal dysfunction, and elevated liver enzymes. These AEs may lead patients to reduce their dosage or even discontinue prophylaxis, thus increasing their risk of PJP (Utsunomiya et al., 2017; Kitazawa et al., 2019). Moreover, some alternative drugs, such as inhaled pentamidine and atovaquone are not as effective as TMP-SMX (Ioannidis et al., 1996; Bozzette et al., 1995). Therefore, it is crucial to avoid discontinuation of TMP-SMX during PJP prophylaxis.

Considering the dose-dependent nature of TMP-SMX-induced AEs (Utsunomiya et al., 2017), it is possible to reduce the incidence of AEs and improve the tolerability of TMP-SMX by reducing the prophylactic dose. Prasad et al. reported that the reduced use of TMP-SMX during standard prophylaxis after renal transplantation did not affect the incidence of PJP and AEs (Prasad et al., 2019). Similarly, Chen et al. demonstrated that using a very small dose of TMP-SMX significantly reduced the incidence of PJP within 6 months while maintaining a favorable safety profile in 1,469 postoperative renal transplantation patients (Chen et al., 2022). In a randomized controlled trial (RCT) of 183 patients with systemic rheumatic diseases, the authors found better drug retention and safety with either 200 mg/40 mg daily (reduced-dose regimen) or gradually increased to 200 mg/40 mg (dose-escalation regimen) compared with a standard prophylactic regimen (Utsunomiya et al., 2020). However, these articles varied in design, population, dosage, and outcomes, which makes the evidence for low-dose TMP-SMX for PJP prophylaxis still unclear (Prasad et al., 2019; Chen et al., 2022; Utsunomiya et al., 2020; Zmarlicka et al., 2015). Therefore, in this study, we aimed to evaluate the efficacy and safety of TMP-SMX at lower than standard prophylactic doses in non-HIV immunocompromised patients.

This study will comprehensively search the literature on this topic and complete it using the meta-analysis method.

## Methods

We conducted this study according to the PRISMA statement (Page et al., 2021) and followed the Cochrane Handbook on Systematic Reviews of Interventions (Supplementary File S1). The protocol has been registered on the International Platform of Registered Systematic Review and Meta-analysis Protocols database (Registration number: INPLASY202440084).

### Search strategy

We searched PubMed, Embase, Wanfang, China National Knowledge Infrastructure, Web of Science, and Cochrane Library databases from their inception until 15 October 2024, for studies reporting the safety and efficacy of using TMP-SMX in PJP prophylaxis in HIV-uninfected patients. The search strategy included MeSH terms and keywords for “prophylaxis,” “TMP-SMX,” “trimethoprim-sulfamethoxazole,” “sulfamethoxazole,” “SMX-TMP,” “*Pneumocystis carinii* pneumonia” and “*Pneumocystis jirovecii* pneumonia,” without any language and study design limitations. The detailed search strategy is summarized in Supplementary File S2. We also screened the reference lists of the selected studies and retrieved reviews to avoid omitting any relevant studies for inclusion. Two authors (H-BH and Y-BZ) conducted independently the literature search and the study selection.

### Study selection

We selected two types of studies for analysis. The first category includes studies reporting discontinuation rates and AEs comparing standard and low doses of TMP-SMX for PJP prophylaxis. The standard dose of TMP-SMX for PJP prophylaxis is one single-strength (80 mg/400 mg) tablet per day or three double-strength (160 mg/800 mg) tablets per week (Taplitz et al., 2018; Tomblyn et al., 2009). Therefore, low-dose TMP-SMX was defined in this study as a total weekly prophylactic dose of less than 6 single-strength, regardless of dosing strategy or frequency of administration. The study design included RCTs and observational studies with two-arm comparisons. The other category includes studies that reported discontinuation rates and AEs in only low-dose of TMP-SMX prophylaxis group, without standard dose comparators. We excluded studies that only enrolled children, studies published in the abstract, conference reports, commentaries, and studies with predefined outcomes data unavailable. In particular, studies that initially used standard or high prophylactic doses of TMP-SMX and then compared patients with and without TMP-SMX discontinuation were also excluded.

### Data extraction and outcomes

Relevant data were extracted from eligible articles, including the study characteristics (author and year, study design, sample size, and country where the study was performed, and follow-up), patient characteristics (age, gender, underlying diseases), dosing regimens (low-dose and standard dose), and predefined outcomes (i.e., discontinuation rates and AEs).

The primary outcome was the overall discontinuation rates during the study period. Secondary outcomes were the incidence of PJP during the follow-up and AEs such as hyponatremia, renal dysfunction (e.g., elevated serum creatinine than baseline, oliguria, or anuria, defined by authors), liver dysfunction (e.g., elevated liver enzymes or bilirubin), thrombocytopenia, fever, rash, anaemia, leukopenia, and hyperpotassemia. Disagreements between the two authors were resolved by consulting a third author (D-XY).

### Quality assessment

H-BH and Y-BZ independently assessed the quality of each included study using the Cochrane Risk of Bias tool for RCTs (Higgins et al., 2011) and the Newcastle-Ottawa Quality Assessment Scale for cohort studies (Stang, 2010). We evaluated publication bias by visual inspection funnel plots when at least 10 studies were included in this meta-analysis. Disagreements were identified and resolved by consensus.

### Statistical analysis

The data were pooled using the DerSimonian and Laird random-effects model for single-arm and controlled studies. For two-arm studies, the results from all relevant studies were combined to estimate the pooled odds ratio (ORs) and associated 95% confidence intervals (CIs) for dichotomous outcomes and estimate mean differences (MD) and 95% CIs for continuous outcomes as effective results.

Relevant studies were pooled to analyze each predefined outcome. To explore the potential influences of the primary outcome (discontinuation rates), we performed sensitivity analyses by pooling studies only focusing on (1) AEs associated discontinuation rate, (2) patients with rheumatic diseases, and (3) mixed patients. Additionally, subgroup analyses were conducted separately by pooling studies based on (1) statistical analysis: fixed-effects mode or random-effects mode; (2) follow-up: ≤6 months or >6 months; (3) study design: RCTs or observation study; (4) sample size: >100 or ≤100; (5) low-dose strategy: dose-reduction or dose dose-escalation; and (6) patients with or without renal dysfunction.

We used the *I* ^2^ statistic to test for heterogeneity, with values of *I* ^2^ < 50% and *I* ^2^ > 50% indicating low and high heterogeneity, respectively (Higgins et al., 2003). A fixed-effect model was used when *I* ^2^ < 50%, and a random-effect model was used when *I* ^2^ > 50%, using the Mantel-Haenszel method. The significance level for *P* values was <0.05. We used Review Manager (version 5.4) for all analyses.

## Results

### Searching results

**FIGURE 1:**
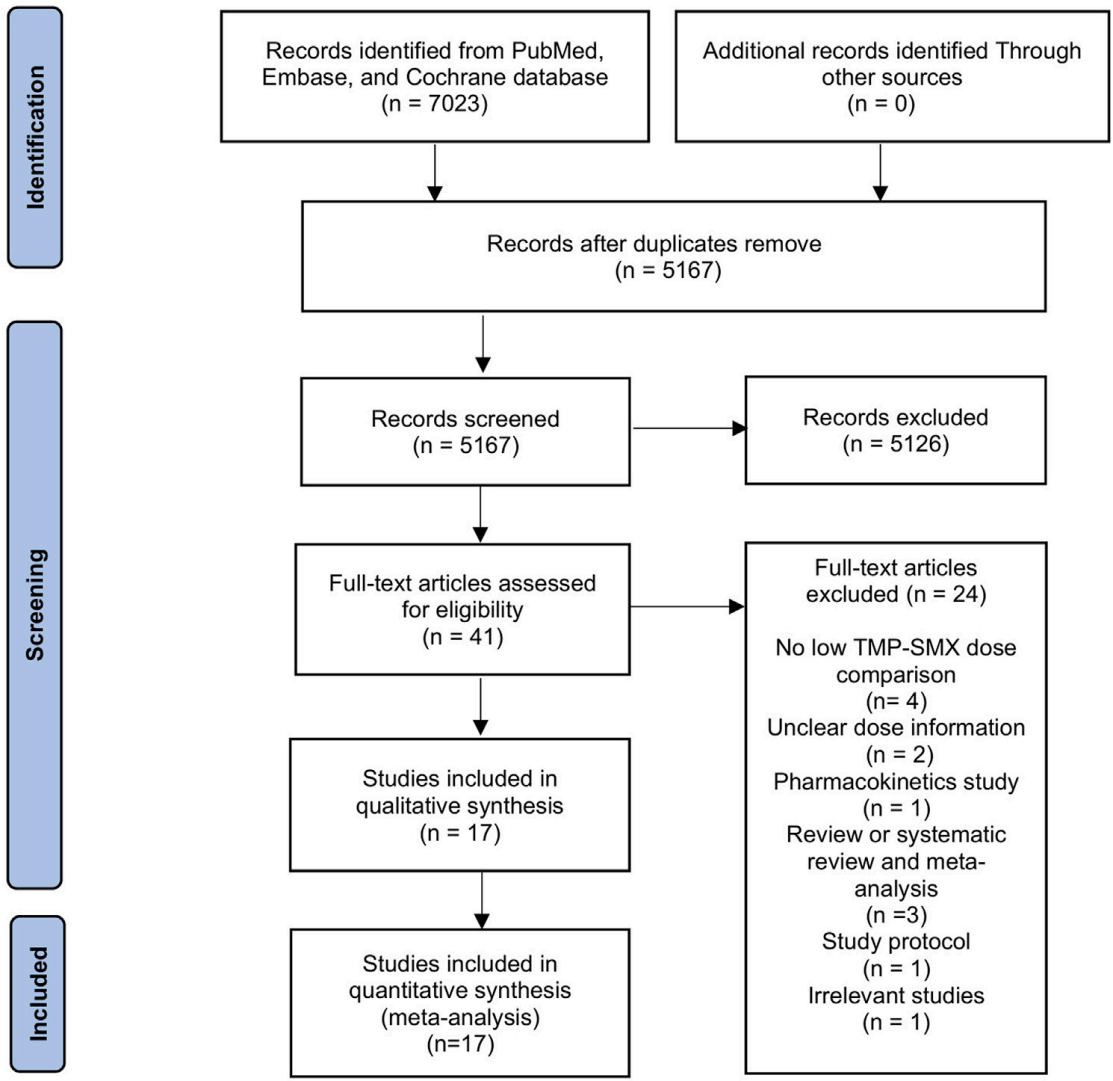
Flow chart of literature selection.

Figure 1 outlines the review process. The original search yielded 7,023 records from the databases and one record from another search source. After de-duplication, 5,167 articles were screened based on title and abstract, leaving 41 for full-text review. Subsequentially, we excluded 24 articles summarized in Supplementary File S3 with reasons for exclusion. Therefore, 17 articles (12 studies with two-arm comparisons and five studies with single arm) were included in the final analysis (Chen et al., 2022; Utsunomiya et al., 2020; Zmarlicka et al., 2015; Harada et al., 2021; Maezawa et al., 2013; Muto et al., 2011; Otani et al., 2021; Peterson et al., 2021; Shimizu et al., 2019; Suyama et al., 2016; Takenaka et al., 2013; Yamamoto et al., 2014; Yamashita et al., 2021; Waki et al., 2021; Yamanaga et al., 2020; Ohmura et al., 2024; Shan et al., 2024).

### Study characteristics and quality assessment

Table 1 and Supplementary File S4 describe the main characteristics of the included studies. These studies were conducted between 2011 and 2024 with 4,896 participants (207 in RCTs and 4,689 in observational studies). Among the included studies, ten compared low-dose with the standard dose of TMP-SMX for PJP prophylaxis (Utsunomiya et al., 2020; Harada et al., 2021; Otani et al., 2021; Shimizu et al., 2019; Suyama et al., 2016; Takenaka et al., 2013; Yamamoto et al., 2014; Yamashita et al., 2021; Waki et al., 2021; Ohmura et al., 2024), three compared a low-dose regimen with no prophylaxis (Chen et al., 2022; Waki et al., 2021; Yamanaga et al., 2020), and the remaining five only contained a low-dose TMP-SMX prophylactic arm (Zmarlicka et al., 2015; Maezawa et al., 2013; Muto et al., 2011; Peterson et al., 2021; Shan et al., 2024). Most included studies focused on specific patient populations, i.e., rheumatic diseases (Utsunomiya et al., 2020; Harada et al., 2021; Suyama et al., 2016; Takenaka et al., 2013; Yamamoto et al., 2014; Waki et al., 2021; Ohmura et al., 2024), hematological malignancy (Muto et al., 2011; Shimizu et al., 2019), and renal transplantation (Chen et al., 2022; Zmarlicka et al., 2015; Peterson et al., 2021; Yamanaga et al., 2020; Shan et al., 2024), while the remaining three recruited mixed populations (Maezawa et al., 2013; Otani et al., 2021; Yamashita et al., 2021). As to the low-dose regimens, two types of strategies were used, with the dose-reduced strategy being the most common (*n* = 15) (Chen et al., 2022; Utsunomiya et al., 2020; Zmarlicka et al., 2015; Harada et al., 2021; Maezawa et al., 2013; Muto et al., 2011; Otani et al., 2021; Peterson et al., 2021; Shimizu et al., 2019; Yamamoto et al., 2014; Yamashita et al., 2021; Waki et al., 2021; Yamanaga et al., 2020; Ohmura et al., 2024; Shan et al., 2024), followed by the dose-escalation strategy (*n* = 3) (Utsunomiya et al., 2020; Suyama et al., 2016; Takenaka et al., 2013).

**TABLE 1:** Characteristics of the included studies.

Study	Study design	Underlying condition, (%)	Sample LD/SD/NP	LD regimen	Control regimen	Mean age, year, LD/SD	Male, %, LD/SD	Follow-up	Predefined outcomes
Ohmura et al. (2024)	R, SC, DA	RD (100)	60/126/0	1 SS (2/w)*	SD: 1 SS (1/d)**	68/55.5	30/26.2	12 M	DR, AE
Harada et al. (2021)	R, SC, DA	RD (100)	75/145/0	1 SS (3–4/w)	SD: 1 SS (1/d) or 2 SS (3/w)	64/58	36.7/34.5	6 M	DR, AE
Yamashita et al. (2021)	R, SC, DA	HM (50), RD (33.3), others (16.7)	36/45/0	<6 SS/w	SD: ≥6 SS/w	67/67	69.4/60	20 M	DR, AE
Otani et al. (2021)	R, SC, DA	ILD (88.4), lung neoplasm (7.0), asthma (4.6)	74/244/0	1 HS (1/d) or 1SS (1/2d)	SD: 1 SS (1/d)	69/68	41.9/66.8	6 M	DR, AE
Utsunomiya et al. (2020)	RCT, MC, DA	RD (100)	59/55/58	1 HS (1/d) or ES#	SD: 1 SS (1/d)	64.7/58.5	35.6/36.2	12 M	DR, AE
Shimizu et al. (2019)	R, SC, DA	HM(100)	33/65/0	1 SS (3/w)	SD: 2 SS (3/w)	-	-	-	AE
Suyama et al. (2016)	R, MC, DA	RD (100)	28/31/0	ES%	SD: 1 SS (1/d)	43.5/37.9	14.3/9.7	3 M	DR, AE
Yamamoto et al. (2014)	RCT, SC, DA	RD (100)	17/18/0	1 SS (2/w)	SD: 1 SS (1/d)	42.9	-	12 M	DR, AE
Takenaka et al. (2013)	R, SC, DA	RD (100)	13/28/0	ES&	SD: 1 SS (1/d)	57.2/63	-	6 M	DR, AE
Waki et al. (2021)	R, SC, DA	RD (100)	167/40/43	1 SS (3/w) or 0.5 SS (1/d)	SD: 2 SS (3/w) or 1 SS (1/d) or NP	76/66.5/75	45.5/35/68.8	6 M	DR, AE
Chen et al. (2022)	R, SC, DA	KT (100)	1193/0/276	0.25 SS (1/d) or 0.25 SS (1/2d)	NP	43/42	62.2/60.5	6 M	DR, AE
Yamanaga et al. (2020)	R, SC, DA	KT (100)	51/0/13	1 SS (3/w)	NP	46.9/48.8	64.7/76.9	1 M	DR, AE
Shan et al. (2024)	R, SC, DA	KT (100)	1763	0.5 SS (1/d)	-	-	-	6 M	AE
Peterson et al. (2021)	R, SC, SA	KT (100)	228	1 SS (3/w)	-	55	63	18 M	DR, AE
Zmarlicka et al. (2015)	R, SC, SA	KT (100)	77	1 SS (3/w)	-	51	64	12 M	DR, AE
Maezawa et al. (2013)	R, SC, SA	RD (57.9), ILD (42.1)	539	1–2 SS (1/d, 2–3/w)	-	59.5	47.7	-	AE
Muto et al. (2011)	R, SC, SA	HM (100)	156	2 SS (1/d, 2/w)	-	42	64.7	30 M	DR, AE

*Two times per week; **Once a day.

ES#, escalation group (ES) started SMX/TMP, 40 mg/8 mg, and the dosage was increased by 40 mg/8 mg weekly up to 200 mg/40 mg and continued for 24 weeks.

ES%, patients in the graded administration group were treated with a 9-day TMP/SMX, graded administration protocol, which was as follows: day 1, 2 mg/0.4 mg; day 2, 4 mg/0.8 mg; day 3, 8 mg/1.6 mg; day 4, 16 mg/3.2 mg; day 5, 40 mg/8 mg; day 6, 80 mg/16 mg; day 7, 160 mg/32 mg, day 8, 320 mg/64 mg; day 9, 400 mg/80 mg.

ES&, patients were started on a daily dose of 10% of 80 mg/400 mg TMP/SMX. The dose was increased by 10% over 3 or more days, and was gradually increased to 100%, and continued with 80 mg/400 mg TMP/SMX.

AE, adverse event; d, day; DA, double-arms studies; DR, discontinuation rate; HM, hematological malignancy; HS, half single-strength tablet (=40 mg/200 mg); ILD, interstitial lung disease; KT, kidney transplant; LD, low-dose reimen; M, month; MC, multi-center; NP, no prophylaxis; R, retrospective; RCT, randomized controlled trials; RD, rheumatic diseases; SA, single-arm study; SC, single-center; SD, standard-dose regimen; SS, single-strength tablet (=80 mg/400 mg), w, week.

We evaluated the risk of bias in each included study using the NOS method and Cochrane risk evaluation tools. The quality of the observational studies was moderate to high (Supplementary File S5), and the risk of bias in RCTs was low in all critical domains (Supplementary File S6). Assessment of publication bias using visually inspecting funnel plots showed no potential publication bias in the included studies (Supplementary File S7).

### Primary outcome

**FIGURE 2:**
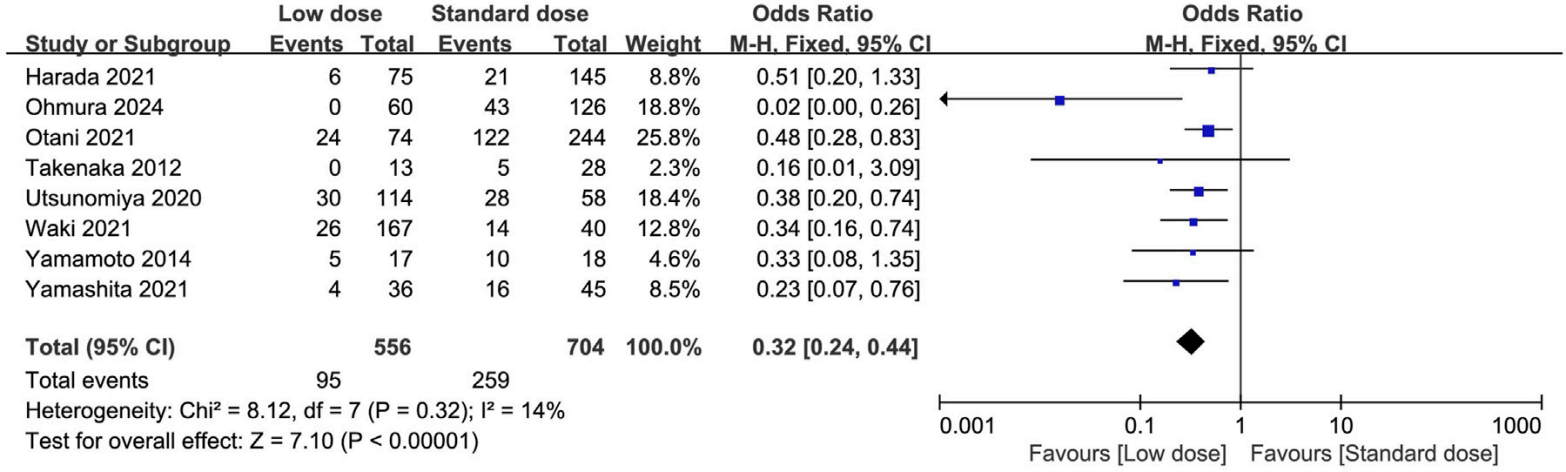
Forest plots of discontinuation rate of low-dose vs. standard dose of trimethoprim-sulfamethoxazole in prophylaxis against *Pneumocystis jirovecii* pneumonia.

Eight studies compared the discontinuation rate between low-dose and standard-dose regimens (Utsunomiya et al., 2020; Harada et al., 2021; Otani et al., 2021; Takenaka et al., 2013; Yamamoto et al., 2014; Yamashita et al., 2021; Waki et al., 2021; Ohmura et al., 2024). Of these, 556 patients received a low-dose prophylactic regimen, and 95 had discontinued (15.98%), compared with 704 patients in the standard dose group, of whom 287 discontinued (40.76%). We found that the low-dose regimen significantly reduced the risks of discontinuation rate compared with the standard dose regimen (OR = 0.32; 95% CI, 0.24–0.44; *I* ^2^ = 14%, *P* < 0.00001) (Figure 2). We performed predefined sensitivity analysis and found consistent results (Table 2), and subsequently excluding any single study from the sensitivity analyses did not significantly change the overall combined OR (all *P* values < 0.00001, and all *I* ^2^ ranged from 8% to 16%). Subgroup analyses were also performed and all of the subgroups based predefined clinical influence factors confirmed a consistent reduction in discontinuation rate in the low-dose prophylactic regimen (Table 2).

**TABLE 2:** Sensitivity and subgroup analyses of low-dose SMX-TMP on discontinuation rate in PJP prophylaxis.

Study characteristics		Studies number	Number of patients	Event in the low-dose group	Event in the standard dose group	Odds ratio (95% CI)	*I* ^2^	*p*
Sensitivity analyses								
	Studies of adverse event associated	6	858	32 of 320	103 of 538	0.39 (0.25, 0.62)	0%	<0.0001
	Studies of rheumatic diseases	6	861	67 of 446	121 of 415	0.28 (0.19, 0.41)	29%	<0.0001
	Studies of mixed patients	2	399	28 of 110	138 of 289	0.42 (0.25, 0.68)	0%	0.0005
Subgroup analyses								
Renal dysfunction	Excluded patients with renal dysfunction	3	288	39 of 167	54 of 121	0.41 (0.27, 0.63)	0%	<0.0001
	Included patients with renal dysfunction	5	972	56 of 389	205 of 583	0.35 (0.18, 0.69)	48%	0.002
Study design	Randomized controlled trial	2	207	35 of 131	38 of 76	0.37 (0.20, 0.68)	0%	0.001
	Non-randomized controlled trial	6	1053	60 of 425	221 of 628	0.31 (0.22, 0.45)	40%	<0.00001
Statistical analysis	Random effects model	8	1260	95 of 556	259 of 704	0.37 (0.26, 0.53)	14%	<0.00001
	Fixed effects model	8	1260	95 of 556	259 of 704	0.32 (0.24, 0.44)	14%	<0.00001
Sample size	≥100	5	1103	86 of 490	228 of 613	0.38 (0.23, 0.63)	43%	0.0001
	<100	3	157	9 of 66	31 of 91	0.25 (0.10, 0.59)	0%	0.002
Follow-up	≤6 months	4	786	56 of 329	162 of 457	0.44 (0.29, 0.65)	0%	<0.0001
	>6 months	4	474	39 of 227	97 of 247	0.24 (0.09, 0.62)	55%	0.003
Low-dose regimen	Reduced dose	6	1047	65 of 429	226 of 618	0.35 (0.21, 0.59)	37%	<0.00001
	Dose-escalation	2	213	30 of 127	33 of 86	0.37 (0.19, 0.70)	0%	0.002

*Calculated according to the control group.

HIV, human immunodeficiency virus-infected; LD, low-dose regimen; RD, renal dysfunction.

**FIGURE 3:**
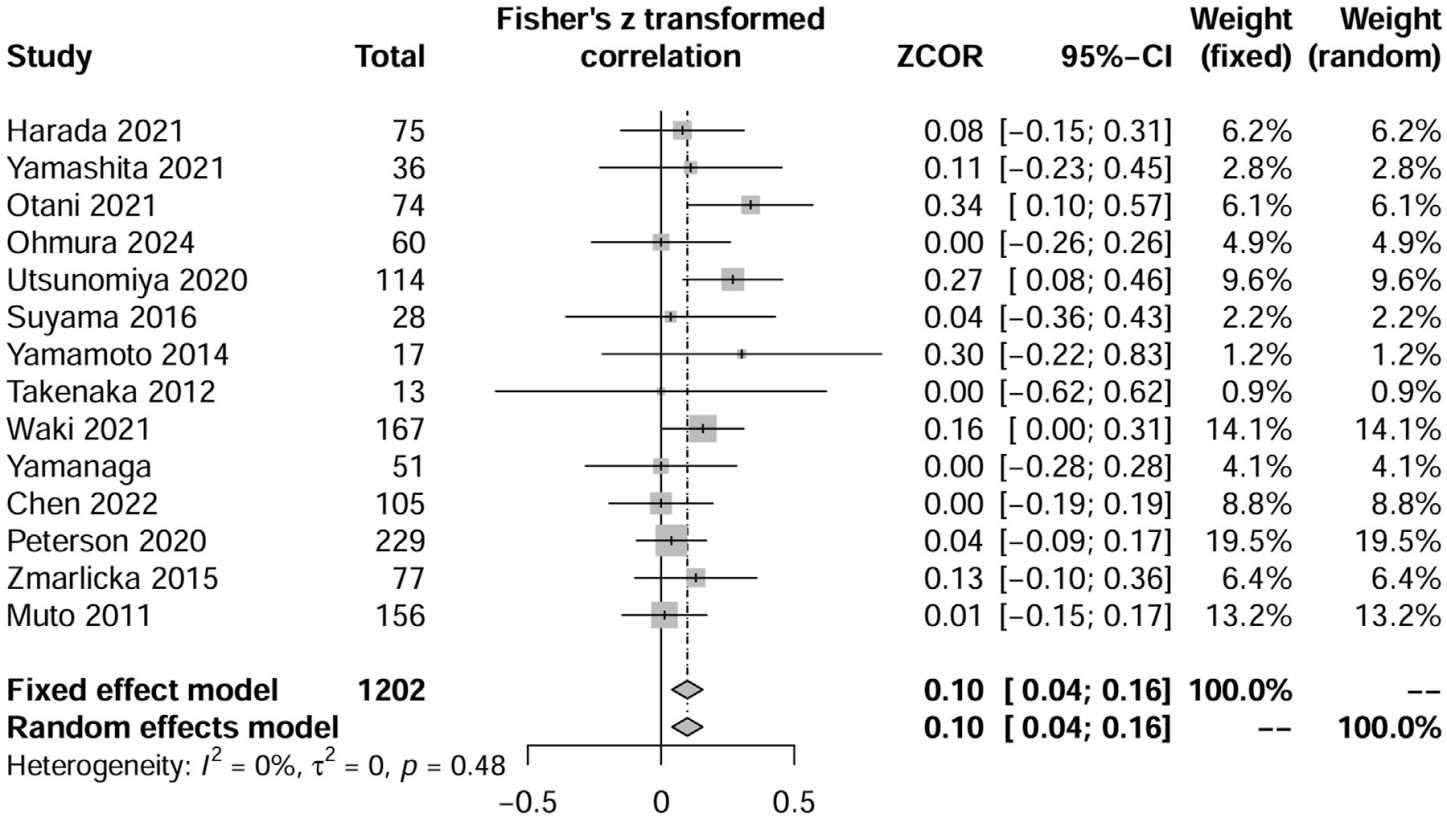
Forest plots of the pooled discontinuation rate from included available studies, including single-arm studies, that reported patients receiving low-dose TMP-SMX in *Pneumocystis jirovecii* pneumonia prophylaxis.

Six additional studies provided data on the low-dose TMP-SMX discontinuation rates (Chen et al., 2022; Zmarlicka et al., 2015; Muto et al., 2011; Peterson et al., 2021; Suyama et al., 2016; Yamanaga et al., 2020). These studies included three two-arm comparative studies of low-dose versus no prevention (Chen et al., 2022; Yamanaga et al., 2020) or standard dose implemented (Suyama et al., 2016), and the other three observational studies reported on only one low-dose TMP-SMX prevention group (Zmarlicka et al., 2015; Muto et al., 2011; Peterson et al., 2021). We combined these studies with the low-dose TMP-SMX groups of the eight studies referred to above (Utsunomiya et al., 2020; Harada et al., 2021; Otani et al., 2021; Takenaka et al., 2013; Yamamoto et al., 2014; Yamashita et al., 2021; Waki et al., 2021; Ohmura et al., 2024). Based on this analysis, we estimated the combined discontinuation rate for patients receiving low-dose TMP-SMX to be 10% (95% CI, 4%–16%), as illustrated in Figure 3.

### Secondary outcomes

**FIGURE 4:**
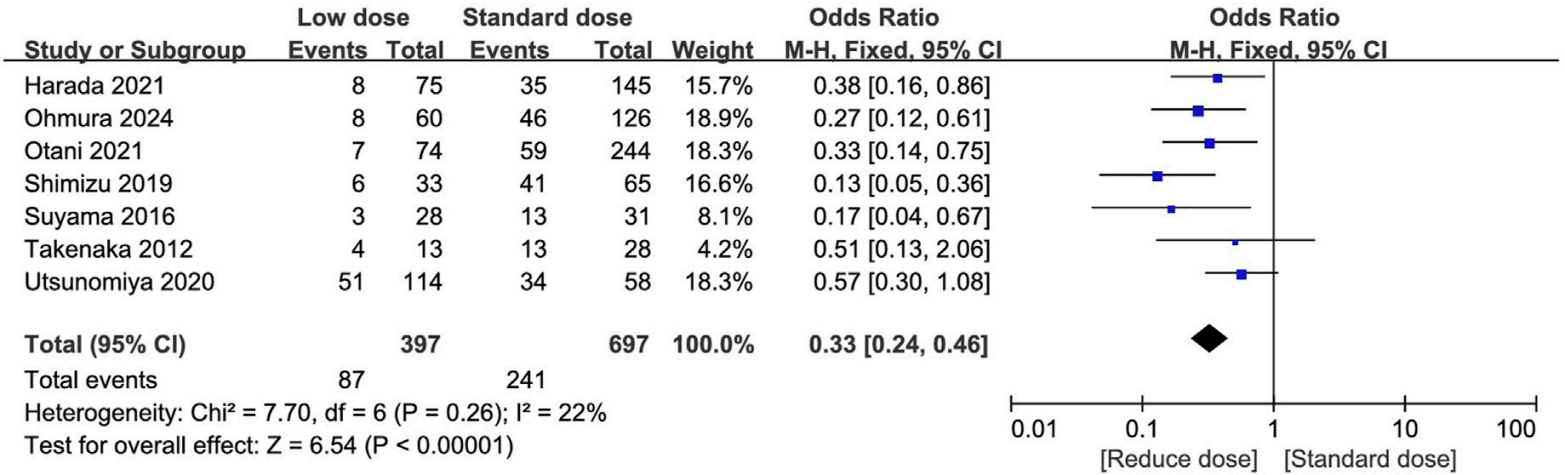
Forest plots of total adverse events of low-dose vs. standard dose of trimethoprim-sulfamethoxazole in prophylaxis against pneumocystis jirovecii pneumonia.

The total AEs and the most frequently occurring AEs (reported in at least three studies) were summarized in Supplementary File S8. Fifteen studies reported the incidence of PJP (Chen et al., 2022; Utsunomiya et al., 2020; Zmarlicka et al., 2015; Harada et al., 2021; Muto et al., 2011; Otani et al., 2021; Peterson et al., 2021; Shimizu et al., 2019; Takenaka et al., 2013; Yamamoto et al., 2014; Yamashita et al., 2021; Waki et al., 2021; Yamanaga et al., 2020; Ohmura et al., 2024; Shan et al., 2024), 12 of which reported no episodes of PJP during follow-up, two studies compared the low- and standard group and found three patients developed PJP (two in the low-dose group and one in the standard-dose group) (Maezawa et al., 2013; Suyama et al., 2016), and the remaining one reported incidence of 1.36% (24/1763) in kidney transplantation patients received low-dose regimen (Shan et al., 2024). Seven studies compared the total AEs between low-dose and standard-dose regimens (Utsunomiya et al., 2020; Harada et al., 2021; Otani et al., 2021; Shimizu et al., 2019; Suyama et al., 2016; Takenaka et al., 2013; Ohmura et al., 2024). The pooled estimates showed that the low-dose regimen significantly reduced the total AEs (OR = 0.33; 95% CI, 0.24–0.46; *I* ^2^ = 22%; *P* < 0.00001; Figure 4) than the standard dose regimen. The most frequently reported AEs were analyzed. The low-dose regimen was associated with a significantly reduced incidence of hyponatremia (OR = 0.24; 95% CI, 0.07–0.78; *I* ^2^ = 0%; *P* = 0.02), and renal dysfunction (OR = 0.39; 95% CI, 0.17–0.86; *I* ^2^ = 0%; *P* = 0.02), liver dysfunction (OR = 0.25; 95% CI, 0.13–0.48; *I* ^2^ = 0%; *P* = 0.0001), thrombocytopenia (OR = 0.41; 95% CI, 0.21–0.81; *I* ^2^ = 0%; *P* = 0.01), fever (OR = 0.17; 95% CI, 0.05–0.53; *I* ^2^ = 10%; *P* = 0.002), and rash (OR = 0.26; 95% CI, 0.14–0.50; *I* ^2^ = 0%; *P* = 0.001). However, the use of the low-dose regimen did not exhibit significant beneficial effect on anaemia (OR = 0.48; 95% CI, 0.13–1.76; *I* ^2^ = 0%; *P* = 0.26), leukopenia (OR = 0.58; 95% CI, 0.24–1.39; *I* ^2^ = 12%; *P* = 0.22), and hyperpotassemia (OR = 0.67; 95% CI, 0.28–1.64; *I* ^2^ = 0%; *P =* 0.38) (Supplementary Figures S1–S9).

## Discussion

In this systematic evaluation, we incorporated 17 studies that met the eligibility criteria. Our main findings indicated as follows. Firstly, compared to the standard dose regimen, the low-dose regimen significantly reduced the discontinuation rate of the initial TMP-SMX protocol (OR = 0.42, 95% CI, 0.29–0.59), and additional subgroup and sensitivity analyses confirmed this result. Secondly, during the study’s observation period, the low-dose regimen significantly decreased the overall incidence of AEs, with improvements observed in all AE types. Specifically, the low-dose regimen significantly reduced the incidences of fever, rash, thrombocytopenia, hyponatremia, elevated serum creatinine, and liver dysfunction. Thirdly, we further evaluated the discontinuation rate and the incidence of various AEs of the low-dose regimen by combining related studies from both single-arm and multi-arm studies to provide a more objective evaluation of this regimen. Given the benefits of this low-dose regimen in terms of efficacy and safety, it is prudent to reconsider the current guidelines and dosing practices for the prophylaxis of PJP using TMP-SMX.

### Compared with previous literature

This study is the first meta-analysis to explore the use of low-dose TMP-SMX for PJP prophylaxis in immunocompromised patients without HIV infection. One previous meta-analysis (Li et al., 2021), including 19 studies with 4,135 patients, confirmed the prophylactic role of the standard dose of TMP-SMX for this patient population. In their finding, the discontinuation rate in the TMP-SMX group was 43.7% (176/403) and is significantly higher than the standard dose group in our study (37.4%, 216/578). This discrepancy may be due to the inclusion of different studies in the two meta-analyses. Moreover, the previous meta-analysis (Li et al., 2021) included the literature for a broad period (1977–2019), which experienced many basic treatment improvements and updates to the PJP guideline. However, despite these factors, our results demonstrated that the low prophylactic dose still significantly reduced the discontinuation rate (19.5%). Subgroup analyses in various clinical settings confirmed the safety of the low prophylactic dose, reinforcing the robustness of our primary findings. Moreover, the results of combined additional single-arm studies suggested a discontinuation rate of 10% for the low-dose regimen (Figure 3). This data, which is closer to the real world, also confirms that low-dose is well tolerated in clinical applications and supports the reliability of our conclusions.

In the above meta-analysis (Li et al., 2021), PJP in the standard dose group was significantly lower than that in the non-prophylactic group (1.3% [14/771] vs. 4.6 [91/1974]). In our meta-analysis, only two included studies reported five patients developing PJP during the prophylactic period, all within the standard dose group, while one study only recruited patients receiving low-dose TMP-SMX and reported 1.36% of patients developed PJP (Shan et al., 2024). This comparison indirectly indicates that the low-dose regimen of TMP-SMX is adequate to achieve its prophylactic purpose.

### Interpretation of our study results

Our results demonstrated the good prophylactic effect of the low-dose regimen. However, some issues need to be considered when interpreting our results. Firstly, the current standard prophylactic regimen of TMP-SMX is based on historical practice rather than being the preferred treatment based on high-quality comparative and dose-exploration studies. However, TMP-SMX has shown its efficacy in several patient populations, including HIV and non-HIV immunocompromised patients (Utsunomiya et al., 2020; Zmarlicka et al., 2015; Page et al., 2021; Li et al., 2021). Several studies have demonstrated superior efficacy in preventing PJP compared to alternative medications (e.g., amisulpride, atovaquone, pentamidine) (Ioannidis et al., 1996; Bozzette et al., 1995). These confirm the value of TMP-SMX in preventing PJP. On the other hand, the fixed standard regimen fails to address the individual differences among PJP patients, such as disparities in etiologies, disease severity, complications, steroid use, and organ functions (Li et al., 2021). Interestingly, two recent meta-analyses have shown that low-dose TMP-SMX (<15–20 mg/kg/d) is as effective as the standard dose regimen (15–20 mg/kg/d) for PJP treatment (Huang et al., 2024; Butler-Laporte et al., 2020). Additionally, the lower dose treatment regimen is associated with better tolerability and fewer adverse events (Huang et al., 2024; Butler-Laporte et al., 2020). Therefore, a lower dose regimen may be sufficient for prophylactic purposes.

Secondly, the clinical benefit of low-dose TMP-SMX for PJP prophylaxis needs to be supported by additional pharmacologic studies. In our study, compared with a low-dose PJP prophylactic regimen, patients receiving the standard dose regimen experienced a higher incidence of dose-dependent AEs, such as rashes, fever, myelosuppression, renal damage, liver dysfunction, and electrolyte imbalances (Utsunomiya et al., 2020; Harada et al., 2021; Otani et al., 2021; Suyama et al., 2016; Takenaka et al., 2013), which suggested an association with increased serum concentrations of TMP-SMX. These findings supported the previous research that high-peak concentrations are related to severe AEs (Klinker et al., 1998). Of note, most of the data on TMP/SMX toxicity comes from studies of HIV-infected adults that may not be translatable to other patient populations. Meanwhile, these toxicities occur even when TMP/SMX is given at low doses, suggesting that toxicity may have a component that depends on the duration of exposure. Moreover, the considerable inter-individual variability in the pharmacokinetics of TMP-SMX may increase the risk of inadequate exposure or toxicity (Brown, 2014). Therefore, more PJP prophylaxis studies are needed in the future to explore the association between dose dependence and exposure period in TMP-SMX and the risk of AEs.

Thirdly, the low-dose prophylactic regimen is safer and more tolerable than the high-dose regimen. In the study by Otani et al. (2021), 19 patients who could not tolerate the standard dose of TMP-SMX due to serious AEs were switched to a half-dose TMP-SMX regimen. Of these 19 patients, 16 (84.2%) could continue PJP prophylaxis. Our study revealed a significant reduction of AE-related discontinuation rate of 8.75% (28/320) in the low-dose regimen compared to 19.14% (103/538) in the high-dose regimen (Utsunomiya et al., 2020; Harada et al., 2021; Otani et al., 2021; Yamamoto et al., 2014; Yamashita et al., 2021). This is mainly due to the reduction in dose-dependent AEs, which makes the low-dose regimen more tolerable. This is particularly important because dose-dependent AEs are usually difficult to manage with supportive medication. When patients stop continuing the prophylaxis regimen, they are at risk of PJP again, especially those who require long-term or lifelong prophylaxis, such as patients who have had lung or intestinal transplantations or have a history of PJP (Ghembaza et al., 2020; Kim et al., 2019). It should be noted that continuing the TMP-SMX prophylaxis has other benefits, such as effectively preventing other opportunistic pathogens like Toxoplasma, gastrointestinal infections, respiratory pathogens, and some urinary tract pathogens (Bodro and Paterson, 2013; Antinori et al., 1995; Carr et al., 1992). However, whether low-dose TMP-SMX can maintain preventive effects on these opportunistic pathogens remains to be confirmed.

### Current literature and future research

First, the definition of a low-dose prophylactic regimen is unclear. Various strategies are being implemented to reduce the dosage, including single strength, half dose, or dose escalation. Our subgroup analyses suggested those low-dose regimens showed benefits in efficacy and safety. However, considering the variability in the pharmacokinetics of TMP-SMX among immunocompromised individuals, future research should integrate patient populations, renal function, and disease severity to establish the optimal threshold for low-dose TMP-SMX prophylaxis.

Second, there is a need to identify which patient population benefits most from the low-dose prophylactic regimen. The study by Otani et al. showed that higher serum creatinine, lower creatinine clearance at baseline, and abnormal liver function were associated with an increased rate of TMP-SMX discontinuation, suggesting that a reduced equivalent dose of TMP-SMX should be considered in these populations (Otani et al., 2021). The study by Chen et al. (2022) demonstrated similar efficacy and fewer AEs in patients with post-transplant PJP treated with a low-dose TMP-SMX regimen compared to those treated with a standard-dose regimen. Maezawa et al. found that TMP-SMX caused more AEs in patients with connective tissue disease than in interstitial lung disease (ILD) patients (7.05% [22/312] vs. 2.64% [9/227]) (Maezawa et al., 2013). However, ILD results in lower antimicrobial concentrations in the lung (Huang et al., 2014), and it remains unclear how much dose is needed to maintain prophylaxis in these patients. In addition, whether low-dose regimens may benefit the critically ill population is not addressed in any of the included studies. Therefore, these questions need to be confirmed by further studies.

### Limitations

To provide a comprehensive review of our study, it is important to acknowledge the limitations. First, most included studies are retrospective, limiting the clarity of causal relationships and should be further validated through prospective trials. Second, some studies have small sample sizes and are conducted in single center, which requires caution in interpreting the results. Third, due to insufficient data, we could not explore some important influencing factors such as the prophylaxis period. Meanwhile, the included studies focus primarily on the prophylaxis of PJP risk within 6 months, and long-term follow-up beyond this period may be required. Fourth, the incidence of some AEs is low and has been assessed in only a few studies, potentially limiting the efficacy evaluation. In addition, the included studies lacked clear standardized definitions of some AEs, which may affect the generalizability of the conclusions. Fifth, the decision to reduce or discontinue TMP-SMX is at the discretion of each physician, which may introduce selection bias for some patients and could affect the discontinuation rate. Finally, most studies involved Asian populations, which may limit the external validity of our study findings across various factors.

## Conclusion

In summary, our analysis demonstrates that a low-dose TMP-SMX PJP prophylactic regimen significantly reduces discontinuation rates in individuals without HIV infection. Furthermore, the low-dose regimen was associated with a significant reduction in AEs. Our study has several limitations, including the study design and the associated high risk of bias, which may have affected the certainty of our findings. However, it is also important to acknowledge the promise of these results, as low-dose TMP-SMX therapy has shown extremely positive results in this patient population. Therefore, future studies based on TMP-SMX concentration monitoring are needed to clarify the optimal reduced prophylactic dose. Meanwhile, large-sample, multicenter, RCTs should be conducted for different PJP-infected populations to confirm our findings.

## Data Availability

The original contributions presented in the study are included in the article/Supplementary material, further inquiries can be directed to the corresponding authors.

## Abbreviations

AEs, adverse events; CI, confidence interval; ICU, intensive care unit; MD, mean difference; OR, odds ratio; PJP, *Pneumocystis jirovecii* pneumonia; RCTs, randomized controlled trials; SD, standard deviations; TMP-SMX, trimethoprim-sulfamethoxazole.
