# Supplementary material for: Low-dose trimethoprim-sulfamethoxazole for prophylaxis of Pneumocystis jirovecii pneumonia in HIV-uninfected patients: a systematic review and meta-analysis
Source: Front Pharmacol. 2025 Jul 15;16:1545436. doi: 10.3389/fphar.2025.1545436 (PMC12303816; doi:10.3389/fphar.2025.1545436)
Supplement: Supplementary file 1 [file Supplementaryfile1.docx]

**Low-dose trimethoprim-sulfamethoxazole for prophylaxis of pneumocystis jirovecii pneumonia: a systematic review and meta-analysis**

**Additional files**

Additional file 1 PRISMA checklist……………………………………………………………………………………………………………………………………………………………………………………………….……………………………………..2

Additional file 2 Search Strategy………………………………………………………………………………………………………………………………………………………………………………………………………………….……………………..5

Additional file 3 Studies needed for review but not included in the current meta-analysis ………………………………………………………………………………..…………………………..………….……………….……..8

Additional file 4 The main characteristics according to PICOS principle of included studies………………………………………………………………………………………………………………………………………….….10

Additional file 5 Assessment of observational studies……………………….……………………………………………….…………………………………………………………………………………………………………………...…….…13

Additional file 6 Assessment of RCTs quality …………….…………………………….…………………………………………………………………………………………………………………………………………………………….....………14

Additional file 7 Funnel plot of comparison: Discontinuation rate…..…………………………………………………………………….…………………………………………………………….…………………………………....………15

Additional file 8 Summary of adverse events of the low-dose group vs. standard group in the included studies………………………………………………….………………………………………………....……….16

Additional File 9 Funnel plot of adverse events……………………………………………………………………………………………………………………………………………………………………………………………………………..…17

**Additional File 1**

**PRISMA 2009 checklist**

| **Section/topic** | **#** | **Checklist item** | **Reported on page #** |
| --- | --- | --- | --- |
| **TITLE** | | |  |
| Title | 1 | Identify the report as a systematic review, meta-analysis, or both. | 1 |
| **ABSTRACT** | | |  |
| Structured summary | 2 | Provide a structured summary including, as applicable: background; objectives; data sources; study eligibility criteria, participants, and interventions; study appraisal and synthesis methods; results; limitations; conclusions and implications of key findings; systematic review registration number. | 2 |
| **INTRODUCTION** | | |  |
| Rationale | 3 | Describe the rationale for the review in the context of what is already known. | 4 |
| Objectives | 4 | Provide an explicit statement of questions being addressed with reference to participants, interventions, comparisons, outcomes, and study design (PICOS). | 4-5 |
| **METHODS** | | |  |
| Protocol and registration | 5 | Indicate if a review protocol exists, if and where it can be accessed (e.g., Web address), and, if available, provide registration information including registration number. | 6 |
| Eligibility criteria | 6 | Specify study characteristics (e.g., PICOS, length of follow-up) and report characteristics (e.g., years considered, language, publication status) used as criteria for eligibility, giving rationale. | 6 |
| Information sources | 7 | Describe all information sources (e.g., databases with dates of coverage, contact with study authors to identify additional studies) in the search and date last searched. | 6 |
| Search | 8 | Present full electronic search strategy for at least one database, including any limits used, such that it could be repeated. | 6-7 and Additional File 2 |
| Study selection | 9 | State the process for selecting studies (i.e., screening, eligibility, included in systematic review, and, if applicable, included in the meta-analysis). | 6-7 |
| Data collection process | 10 | Describe method of data extraction from reports (e.g., piloted forms, independently, in duplicate) and any processes for obtaining and confirming data from investigators. | 7 |
| Data items | 11 | List and define all variables for which data were sought (e.g., PICOS, funding sources) and any assumptions and simplifications made. | 7 |
| Risk of bias in individual studies | 12 | Describe methods used for assessing risk of bias of individual studies (including specification of whether this was done at the study or outcome level), and how this information is to be used in any data synthesis. | 7 |
| Summary measures | 13 | State the principal summary measures (e.g., risk ratio, difference in means). | 7 |
| Synthesis of results | 14 | Describe the methods of handling data and combining results of studies, if done, including measures of consistency (e.g., I^2^) for each meta-analysis. | 7-8 |

| Risk of bias across studies | 15 | Specify any assessment of risk of bias that may affect the cumulative evidence (e.g., publication bias, selective reporting within studies). | 8 |
| --- | --- | --- | --- |
| Additional analyses | 16 | Describe methods of additional analyses (e.g., sensitivity or subgroup analyses, meta-regression), if done, indicating which were pre-specified. | 8 |
| **RESULTS** | | |  |
| Study selection | 17 | Give numbers of studies screened, assessed for eligibility, and included in the review, with reasons for exclusions at each stage, ideally with a flow diagram. | 9 and Figure 1, Additional File 3 |
| Study characteristics | 18 | For each study, present characteristics for which data were extracted (e.g., study size, PICOS, follow-up period) and provide the citations. | 9  Table 1, |
| Risk of bias within studies | 19 | Present data on risk of bias of each study and, if available, any outcome level assessment (see item 12). | 9-10 |
| Results of individual studies | 20 | For all outcomes considered (benefits or harms), present, for each study: (a) simple summary data for each intervention group (b) effect estimates and confidence intervals, ideally with a forest plot. | 10 |
| Synthesis of results | 21 | Present results of each meta-analysis done, including confidence intervals and measures of consistency. | 10-11 |
| Risk of bias across studies | 22 | Present results of any assessment of risk of bias across studies (see Item 15). | 10，Additional File 4 |
| Additional analysis | 23 | Give results of additional analyses, if done (e.g., sensitivity or subgroup analyses, meta-regression [see Item 16]). | 10-11  Additional File 5-6 |
| **DISCUSSION** | | |  |
| Summary of evidence | 24 | Summarize the main findings including the strength of evidence for each main outcome; consider their relevance to key groups (e.g., healthcare providers, users, and policy makers). | 12-16 |
| Limitations | 25 | Discuss limitations at study and outcome level (e.g., risk of bias), and at review-level (e.g., incomplete retrieval of identified research, reporting bias). | 16 |
| Conclusions | 26 | Provide a general interpretation of the results in the context of other evidence, and implications for future research. | 17 |
| **FUNDING** | | |  |
| Funding | 27 | Describe sources of funding for the systematic review and other support (e.g., supply of data); role of funders for the systematic review. | 20 |

**Appendix 2**

**Search Strategy**

Database: 5 databases

Search completed October 15, 2024

----------------------------------------------------------------------------------------------------------------------

**PubMed**

#1 (Pneumocystis*[Title/Abstract] OR (PJP, pneumonia[Title/Abstract])) OR (PCP, pneumonia[Title/Abstract])) OR ("Pneumonia, Pneumocystis"[Mesh]))

#2 (((((trimethoprim/sulfamethoxazole[Title/Abstract]) OR (TMP-SMX[Title/Abstract])) OR (TMP/SMX[Title/Abstract])) OR ("Trimethoprim, Sulfamethoxazole Drug Combination"[Mesh])) OR (co-trimoxazole[Title/Abstract]))

#3 #1 AND #2

**Embase**

No. Query

#12 #4 AND #11

#11 #5 OR #6 OR #7 OR #8 OR #9 OR #10

#10 'cotrimoxazole'/exp

#9 'co-trimoxazole':ab,ti AND ([embase]/lim OR [medline]/lim)

#8 'smx-tmp':ab,ti AND ([embase]/lim OR [medline]/lim)

#7 'tmp/smx':ab,ti AND ([embase]/lim OR [medline]/lim)

#6 'tmp-smx':ab,ti AND ([embase]/lim OR [medline]/lim)

#5 'trimethoprim/sulfamethoxazole':ab,ti AND ([embase]/lim OR [medline]/lim)

#4 #1 OR #2 OR #

#3 'pneumocystis pneumonia'/exp

#2 'pneumocystis':ab,ti AND ([embase]/lim OR [medline]/lim)

#1 'pneumocystis jirovecii pneumonia':ab,ti AND ([embase]/lim OR [medline]/lim)

**Cochrane library**

ID Search#1

#1 ("pneumocystis jirovecii pneumonia"):ti,ab,kw (Word variations have been searched)

#2 ("pneumocystis"):ti,ab,kw (Word variations have been searched)

#3 ("Pneumocystis carinii pneumonia"):ti,ab,kw (Word variations have been searched)

#4 ("pneumocystis pneumonia"):ti,ab,kw (Word variations have been searched)

#5 #1 OR #2 OR #3 OR #4

#6 ("trimethoprim/sulfamethoxazole"):ti,ab,kw (Word variations have been searched)

#7 ("co-trimoxazole"):ti,ab,kw (Word variations have been searched)

#8 ("tmp-smx"):ti,ab,kw (Word variations have been searched)

#9 ("smx-tmp"):ti,ab,kw (Word variations have been searched)

#10 #6 OR #7 OR #8 OR #9

#11 #5 AND #10

**Web of Science**

1. trimethoprim/sulfamethoxazole [topic]

2. TMP-SMX [topic]

3 TMP/SMX [topic]

4. SMP-TMP [topic]

5. co-trimoxazole [topic]

6. or/1-5

7. pneumocystis jirovecii pneumonia [topic]

8. Pneumocystis carinii pneumonia [topic]

9. pneumocystosis [topic]

10. PJP [topic]

11. PCP [topic]

12. Pneumocystis pneumonia [topic]

13. or/7-12

14. 6 and 13

**Wanfang database**

主题：（肺孢子菌肺炎 or 卡氏肺孢子菌肺炎 or 卡氏肺囊虫病 or 肺孢子菌 or 肺孢子虫肺炎 or 肺囊虫肺炎 or 免疫缺陷性肺炎）and 主题：（磺胺 or 复方磺胺 or 对氨基苯磺酰胺 or 磺胺甲噁唑 or 甲氧苄啶）

**China National Knowledge Infrastructure**

TKA=(“肺孢子菌肺炎” + “卡氏肺孢子菌肺炎” + “卡氏肺囊虫病” + “肺孢子菌” + “肺孢子虫肺炎“ + “肺囊虫肺炎” + “免疫缺陷性肺炎”）and TKA=(“磺胺” or “复方磺胺” or “对氨基苯磺酰胺”+ “磺胺甲噁唑” + “甲氧苄啶”)

**Additional File 3**

**Table. Studies needed for full-reviewed but not included in the current meta-analysis (n=24 articles).**

| No | Study | Reason of exclusion |
| --- | --- | --- |
| 1 | Skoloda D, Newman M, Norman H, et al. Impact of Prophylactic Trimethoprim-Sulfamethoxazole on Clearance of High-Dose Methotrexate in Adult Patients. JCO Oncol Pract. 2024 Feb 21:OP2300792. | A |
| 2 | Alshehri S, Alghuraybi R, Ayoub E, eet al. Evaluation of Weight-Based Co-trimoxazole Dosing in a Saudi Tertiary Hospital. Cureus. 2023 Oct 20;15(10):e47400. | B |
| 3 | Haseeb A, Abourehab MAS, Almalki WA, et al. Trimethoprim-Sulfamethoxazole (Bactrim) Dose Optimization in Pneumocystis jirovecii Pneumonia (PCP) Management: A Systematic Review. Int J Environ Res Public Health. 2022 Feb 28;19(5):2833. | C |
| 4 | Sonomoto K, Tanaka H, Nguyen TM, et al. Prophylaxis against pneumocystis pneumonia in rheumatoid arthritis patients treated with b/tsDMARDs: insights from 3787 cases in the FIRST registry. Rheumatology (Oxford). 2022 May 5;61(5):1831-1840. | A |
| 5 | Jinno S, Akashi K, Onishi A, et al. Comparative effectiveness of trimethoprim-sulfamethoxazole versus atovaquone for the prophylaxis of pneumocystis pneumonia in patients with connective tissue diseases receiving prolonged high-dose glucocorticoids. Rheumatol Int. 2022 Aug;42(8):1403-1409. | A |
| 6 | McAleese J, Mooney L, Walls GM. Reducing the Risk of Death From Pneumocystis jirovecii Pneumonia After Radical Radiation Therapy to the Lung. Clin Oncol (R Coll Radiol). 2021 Dec;33(12):780-787. | F |
| 7 | Honda N, Tagashira Y, Kawai S, et al. Reduction of Pneumocystis jirovecii pneumonia and bloodstream infections by trimethoprim-sulfamethoxazole prophylaxis in patients with rheumatic diseases. Scand J Rheumatol. 2021 Sep;50(5):365-371. | A |
| 8 | Pereda CA, Nishishinya-Aquino MB, et al. Is cotrimoxazole prophylaxis against Pneumocystis jirovecii pneumonia needed in patients with systemic autoimmune rheumatic diseases requiring immunosuppressive therapies? Rheumatol Int. 2021 Aug;41(8):1419-1427. | C |
| 9 | Ganu SA, Mathew AJ, Nadaraj A, et al. Cotrimoxazole prophylaxis prevents major infective episodes in patients with systemic lupus erythematosus on immunosuppressants: A non-concurrent cohort study. Lupus. 2021 May;30(6):893-900. | A |
| 10 | Raso S, Napolitano M, Arrigo G, et al. Antimicrobial prophylaxis in patients with immune thrombocytopenia treated with rituximab: a retrospective multicenter analysis. Ann Hematol. 2021 Mar;100(3):653-659. | A |
| 11 | Park JW, Curtis JR, Kim MJ, et al. Pneumocystis pneumonia in patients with rheumatic diseases receiving prolonged, non-high-dose steroids-clinical implication of primary prophylaxis using trimethoprim-sulfamethoxazole. Arthritis Res Ther. 2019 Sep 14;21(1):207. | A |
| 12 | Gkirkas K, Stamouli M, Thomopoulos T, et al. Low-Dose Cotrimoxazole Administered in Hematopoietic Stem Cell Transplant Recipients as Prophylaxis for Pneumocystis jirovecii Pneumonia Is Effective in Prevention of Infection due to Nocardia. Biol Blood Marrow Transplant. 2019 Sep;25(9):e298-e299. | A |
| 13 | Prasad GVR, Beckley J, Mathur M, et al. Safety and efficacy of prophylaxis for Pneumocystis jirovecii pneumonia involving trimethoprim-sulfamethoxazole dose reduction in kidney transplantation. BMC Infect Dis. 2019 Apr 5;19(1):311. | B |
| 14 | Abe Y, Fujibayashi K, Nishizaki Y, et al. Conventional-dose Versus Half-dose Sulfamethoxazole-trimethoprim for the Prophylaxis of Pneumocystis Pneumonia in Patients with Systemic Rheumatic Disease: A Non-blind, Randomized Controlled Trial. Acta Med Okayama. 2019 Feb;73(1):85-89. | D |
| 15 | Coppens R, Yang J, Ghosh S, Gill J, Chambers C, Easaw JC. Evaluation of laboratory disturbance risk when adding low-dose cotrimoxazole for PJP prophylaxis to regimens of high-grade glioma patients taking RAAS inhibitors. J Oncol Pharm Pract. 2019 Sep;25(6):1366-1373. | A |
| 16 | Park JW, Curtis JR, Moon J, Song YW, Kim S, Lee EB. Prophylactic effect of trimethoprim-sulfamethoxazole for pneumocystis pneumonia in patients with rheumatic diseases exposed to prolonged high-dose glucocorticoids. Ann Rheum Dis. 2018 May;77(5):644-649. | A |
| 17 | Watts CS, Sciasci JN, Pauley JL, Panetta JC, Pei D, Cheng C, Christensen CM, Mikkelsen TS, Pui CH, Jeha S, Relling MV. Prophylactic Trimethoprim-Sulfamethoxazole Does Not Affect Pharmacokinetics or Pharmacodynamics of Methotrexate. J Pediatr Hematol Oncol. 2016 Aug;38(6):449-52. | E |
| 18 | Higashioka K, Niiro H, Yoshida K, Oryoji K, Kamada K, Mizuki S, Yokota E. Renal Insufficiency in Concert with Renin-angiotensin-aldosterone Inhibition Is a Major Risk Factor for Hyperkalemia Associated with Low-dose Trimethoprim-sulfamethoxazole in Adults. Intern Med. 2016;55(5):467-71. | B |
| 19 | Chew LC, Maceda-Galang LM, Tan YK, Chakraborty B, Thumboo J. Pneumocystis jirovecii pneumonia in patients with autoimmune disease on high-dose glucocorticoid. J Clin Rheumatol. 2015 Mar;21(2):72-5. | A |
| 20 | Horwedel TA, Bowman LJ, Saab G, Brennan DC. Benefits of sulfamethoxazole-trimethoprim prophylaxis on rates of sepsis after kidney transplant. Transpl Infect Dis. 2014 Apr;16(2):261-9. | A |
| 21 | Clajus C, Kühn-Velten WN, Schmidt JJ, Lorenzen JM, Pietsch D, Beutel G, Kielstein JT. Cotrimoxazole plasma levels, dialyzer clearance and total removal by extended dialysis in a patient with acute kidney injury: risk of under-dosing using current dosing recommendations. BMC Pharmacol Toxicol. 2013 Apr 3;14:19. | E |
| 22 | Vananuvat P, Suwannalai P, Sungkanuparph S, Limsuwan T, Ngamjanyaporn P, Janwityanujit S. Primary prophylaxis for Pneumocystis jirovecii pneumonia in patients with connective tissue diseases. Semin Arthritis Rheum. 2011 Dec;41(3):497-502. | B |
| 23 | Li R, Tang Z, Liu F, Yang M. Efficacy and safety of trimethoprim-sulfamethoxazole for the prevention of pneumocystis pneumonia in human immunodeficiency virus-negative immunodeficient patients: A systematic review and meta-analysis. PLoS One. 2021 Mar 25;16(3):e0248524. | C |
| 24 | Giullian JA, Cavanaugh K, Schaefer H. Lower risk of urinary tract infection with low-dose trimethoprim/sulfamethoxazole compared to dapsone prophylaxis in older renal transplant patients on a rapid steroid-withdrawal immunosuppression regimen. Clin Transplant. 2010 Sep-Oct;24(5):636-42. | A |

A = no low TMP-SMX dose comparison; B = unclear dose information; C = review or systematic review and meta-analysis; D = study protocol; E = pharmacokinetics study; F = irrelevant studies.

**Additional File 4**

The inclusion criteria were according to the PICOS principle as follows:

(1) Population: *pneumocystis jirovecii pneumonia* in patients without HIV infection;

(2) Intervention: low-dose trimethoprim-sulfamethoxazole for prophylaxis, defined in this study as a total weekly prophylactic dose of less than 6 single-strength, regardless of dosing strategy or frequency of administration.

(3) Comparators: standard dose of TMP-SMX for PJP prophylaxis, defined as one single-strength (80 mg/400 mg) tablet per day or three double-strength (160 mg/800 mg) tablets per week

(5) Outcomes: The primary outcome was the overall discontinuation rates during the study period. Secondary outcomes were the incidence of PJP during the follow-up and adverse events.

(4) Study design: observation or randomized controlled trials.

**The main characteristics according to PICOS principle of included studies**

| Study | Population  (%) | Intervention  (LD regimen) | Control | Outcomes | Study design |
| --- | --- | --- | --- | --- | --- |
| Ohmura 2024 [37] | RD (100) | 1 SS (2/w)* | SD: 1 SS (1/d)** | 1. The discontinuation rates of SMX/TMP were significantly lower in the low-dose group than those in the conventional dose group after adjusting for clinical characteristics, especially liver dysfunction (P < 0.001). 2. The rate of severe ADRs in the low-dose group was significantly lower than that in the conventional-dose group before adjusting for clinical characteristics (P < 0.05) but was not significantly different in the adjusted analysis. | R, SC, DA |
| Harada 2021 [25] | RD (100) | 1 SS (3-4/w) | SD: 1 SS (1/d) or 2 SS (3/w) | 1. Compared to the dose-reduction group, the conventional group had a significantly high frequency of AEs (10.7% vs. 24.1%; p = .017). 2. The rate of discontinuing SMX/TMP was not significantly different (8.0% vs. 14.5%; p = .165) between the two regimens. 3. The conventional SMX/TMP dose and renal dysfunction were associated with AEs in multivariate analysis | R, SC, DA |
| Yamashita 2021 [34] | HM (50), RD (33.3), others (16.7) | < 6 SS /w | SD: ≥ 6 SS /w | 1. No patients in either group developed PCP during the observation period. 2. The yearly cumulative incidence of discontinuation was 12.1% (95% confidence interval [CI]: 0.027-0.29) in the low-dose group and 35.6% (95% CI: 0.20-0.52) in the standard-dose group (P = 0.019). 3.The adjusted hazard ratio of the low-dose group compared to standard-dose group was 0.18 (95% CI: 0.04-0.86, P = 0.032). | R, SC, DA |
| Otani 2021 [28] | ILD (88.4), lung neoplasm (7.0), asthma (4.6) | 1 HS (1/d) or 1SS (1/2d) | SD: 1 SS (1/d) | 1. PcP did not occur in either group. 2. The univariate and multivariate Cox proportional hazards models revealed that the SS dosage and renal function (e.g. serum creatinine and creatinine clearance) were independently associated with prophylaxis discontinuation. 3. At 24 weeks, the HS group presented significantly lower discontinuation rates than the SS group (P = 0.019, log-rank test). | R, SC, DA |
| Utsunomiya 2020 [19] | RD (100) | 1 HS (1/d) or ES# | SD: 1 SS (1/d) | 1. PCP did not develop in any of the patients by week 52. 2. The overall discontinuation rate was significantly lower in HS than in SS (22.7 vs 47.2%, P = 0.004). The discontinuation rates attributable to adverse events were significantly lower in HS (19.1%, P = 0.007) and ES (20.3%, P = 0.007) than in SS (41.8%). 3. The IRs of adverse events requiring SMX/TMP dose reduction before week 52 differed among the three groups, with a significantly higher IR in SS than in HS or ES (P = 0.007). | RCT, MC, DA |
| Shimizu 2019 [30] | HM(100) | 1 SS (3/w) | SD: 2 SS (3/w) | 1.No patient developed PCP. 2. Patients in group B exhibited a significantly higher incidence of AEs (18.2% vs. 63.1%; p<0.05) and increased AST (6.1% vs. 26.6%; p<0.05), compared with group A. | R, SC, DA |
| Suyama 2016 [31] | RD (100) | ES% | SD: 1 SS (1/d) | 1. The incidence of ADRs was 41.9% in the non-graded administration group, vs. 10.7% in the graded administration group (p = 0.009). 2. The rate of high fever, liver function test (LFT) abnormality, shortness of breath, and hospitalization were reduced in upfront graded administration group | R, MC, DA |
| Yamamoto 2014 [33] | RD (100) | 1 SS (2/w) | SD: 1 SS (1/d) | 1.The incidence of ADRs was 41.9% in the non-graded administration group, vs. 10.7% in the graded administration group (p = 0.009). 2. The rate of high fever, liver function test (LFT) abnormality, shortness of breath, and hospitalization were reduced in upfront graded administration group. | RCT, SC, DA |
| Takenaka 2012 [32] | RD (100) | ES& | SD: 1 SS (1/d) | 1. In the dose-escalation group, the retention rate was 100 % at 6 months. In the routine group, 5 patients discontinued TMP/SMX; the retention rate was 82.1 %. 2. The retention rate when taking a daily dose of 50 % or more of SS TMP/SMX, or 1 SS tablet thrice weekly, was significantly higher in the dose-escalation group (100 versus 71.4 %, P = 0.032). 3. No PCP was observed in the dose-escalation group; however, 1 patient in the routine group, who had discontinued TMP/SMX, developed PCP. 4. The rate of adverse effects was less, although nonsignificant, in the | R, SC, DA |
| Waki 2021 [35] | RD (100) | 1 SS (3/w) or 0.5 SS (1/d) | SD: 2 SS (3/w) or 1 SS (1/d) or NP | The reduced TMP/SMX exposure group had a significant protective effect against early severe infections (standard-dose group versus no TMP/SMX group: hazard ratio [HR] 0.393, 95% confidence interval [CI]: 0.139–1.11, p=0.077; reduced-dose group versus no TMP/SMX group: HR 0.418, 95%CI: 0.216–0.807, p=0.009), even when considering time-dependent changes. | R, SC, DA |
| Chen 2022 [18] | KT(100) | 0.25 SS (1/d) or 0.25 SS (1/2d) | NP | 1. The 276 recipients in the non-prophylaxis group had 124.92 person-years of follow-up, during which PJP occurred in 29 patients, for an incidence rate of 23.21 (95% confidence interval 15.76-32.72) per 100 person-years. The TMP-SMX daily group and the TMP-SMX every other day group had 524.89 and 62.07 person-years of follow-up, respectively, with no occurrence of PJP. 2. There was no significant difference among the three groups in changes in renal and liver function ( P > 0.05, respectively). | R, SC, DA |
| Yamanaga 2020 [36] | KT (100) | 1 SS (3/w) | NP | 1. All patients completed re-administration without adverse events. 2. Serum creatinine increased significantly in the re-administration group (1.40 0.64 mg/dL to 1.48 0.70 mg/dL, P < .01) while not in the control group. | R, SC, DA |
| Shan 2024 [38] | KT (100) | 0.5 SS (1/d) | - | 24 patients were infected with PJP. The overall morbidity of PJP infection in our study was 1.36%. | R, SC, DA |
| Peterson 2020 [29] | KT(100) | 1 SS (3/w) | - | 1.Long-term maintenance was high with 192/237 (81.0 %) patients remaining on prophylaxis at 18-months. 2. Of the remaining 45 patients who initiated prophylaxis, 36/237 (15.2%) were non-adherent and 9/237 (3.8%) discontinued prophylaxis by 18-months. | R, SC, SA |
| Zmarlicka 2015 [20] | KT(100) | 1 SS (3/w) | - | A low-dose prophylactic SMX/TMP regimen of 1 single-strength tablet 3 times weekly is well tolerated. Discontinuation rates were lower than other rates reported for higher-dose regimens. | R, SC, SA |
| Maezawa 2012 [26] | RD (57.9), ILD(42.1) | 1-2 SS (1/d, 2-3/w) | - | 1. AEs occurred in 22 of 312 (7.05 %) CTD patients, while only six of 227 (2.64 %) pulmonary disease patients developed AEs. 2. The incidence of AEs was significantly higher in SLE (11.0 %) and mixed connective tissue disease (MCTD) (33.3 %)patients than in other CTD patients. AEs occurred in 25 % of patients with anti-RNP antibody. | R, SC, SA |
| Muto 2011 [27] | HM (100) | 2 SS (1/d, 2/w) | - | Only one patient with AML (0.6%), who had a leukemia relapse on day 68, developed late-onset pneumocystis pneumonia at 194 days after discontinuation of trimethoprim–sulfamethoxazole. | R, SC, SA |

*Two times per week; **Once a day.

ES#: escalation group (ES) started SMX/TMP 40 mg/8 mg, and the dosage was increased by 40 mg/8 mg weekly up to 200 mg/40 mg and continued for 24 weeks.

ES%: patients in the graded administration group were treated with a 9-day TMP/SMX graded administration protocol, which was as follows: day 1, 2mg/0.4mg; day 2, 4mg/0.8mg;

day 3, 8mg/1.6mg; day 4, 16mg/3.2mg; day 5, 40mg/8mg; day 6, 80mg/16mg; day 7, 160mg/32mg, day 8, 320mg/64mg; day 9, 400mg/80mg.

ES&: patients were started on a daily dose of 10 % of 80 mg/400 mg TMP/SMX. The dose was increased by 10 % over 3 or more days, and was gradually increased to 100 %, and continued with 80 mg/400 mg TMP/SMX.

AE = adverse event; d = day; DA = double-arms studies; DR = discontinuation rate; HM = hematological malignancy; HS = half single-strength tablet (=40 mg/200 mg); ILD = interstitial lung disease; KT = kidney transplant; LD = low-dose reimen; M = month; MC = multi-center; NP = no prophylaxis; R = retrospective; RCT = randomized controlled trials; RD = rheumatic diseases; SA = single-arm study; SC = single-center; SD = standard-dose regimen; SS = single-strength tablet (=80 mg/400 mg), w = week.

**Additional File 5**

**Assessment of study quality**

**Table S6: Quality assessment and overall risk of bias of included observational studies with comparations.**

| First author / year | Patient selection | | | | Comparability | Outcome | | | Risk of bias |
| --- | --- | --- | --- | --- | --- | --- | --- | --- | --- |
|  | Representation of the exposed cohort | Selection of the non-exposed cohort | Ascertainment of exposure | Outcome of  interest not  present at start | Comparability of cohorts on the basis of the design or analysis | Assessment  of outcome | Was follow-up long enough for outcomes to occur | Adequacy of follow up of cohorts |  |
| Harada 2021 [25] | ★ | ★ | ★ | ☆ | ☆★ | ★ | ★ | ★ | 7 |
| Ohmura 2024 [37] | ★ | ★ | ★ | ☆ | ★★ | ★ | ★ | ★ | 8 |
| Yamashita 2021 [34] | ★ | ★ | ★ | ☆ | ★★ | ★ | ★ | ★ | 8 |
| Otani 2021 [28] | ★ | ★ | ★ | ☆ | ★★ | ★ | ★ | ★ | 8 |
| Shimizu 2019 [30] | ★ | ★ | ★ | ☆ | ★★ | ★ | ★ | ★ | 8 |
| Suyama 2016 [31] | ★ | ★ | ★ | ☆ | ☆☆ | ★ | ★ | ★ | 6 |
| Takenaka 2012 [32] | ★ | ★ | ★ | ☆ | ☆★ | ★ | ★ | ★ | 7 |
| Waki 2021 [35] | ★ | ★ | ★ | ☆ | ★★ | ★ | ★ | ★ | 8 |
| Chen 2022 [18] | ★ | ★ | ★ | ☆ | ☆★ | ★ | ★ | ★ | 7 |
| Yamanaga [36] | ★ | ★ | ★ | ☆ | ☆★ | ★ | ★ | ★ | 7 |
| Shan 2024 [38] | ★ | ★ | ★ | ☆ | ☆☆ | ★ | ★ | ★ | 6 |
| Peterson 2020 [29] | ★ | ★ | ★ | ☆ | ☆☆ | ★ | ★ | ★ | 6 |
| Zmarlicka 2015 [20] | ★ | ★ | ★ | ☆ | ☆★ | ★ | ★ | ★ | 7 |
| Maezawa 2012 [26] | ★ | ★ | ★ | ☆ | ☆★ | ★ | ★ | ★ | 7 |
| Muto 2011 [27] | ★ | ★ | ★ | ☆ | ☆★ | ★ | ★ | ★ | 7 |

**Additional File 6**

**Quality assessment and overall risk of bias of included RCTs.**

| Study | Random sequence generation(selection bias) | Allocation concealment (selection bias) | Blinding of participants and personnel (performance bias) | Blinding of outcome assessment (detection bias) | Incomplete outcome data (attrition bias) | Selective reporting (reporting bias) | Other bias |
| --- | --- | --- | --- | --- | --- | --- | --- |
| Utsunomiya 2020 | Low risk | Low risk | High risk | High risk | Low risk | Low risk | Low risk |
| Yamamoto 2014 | Low risk | Low risk | Unclear risk | Unclear risk | Low risk | Low risk | Low risk |

**Additional File 7**

**Funnel plot of comparison: Discontinuation rate**


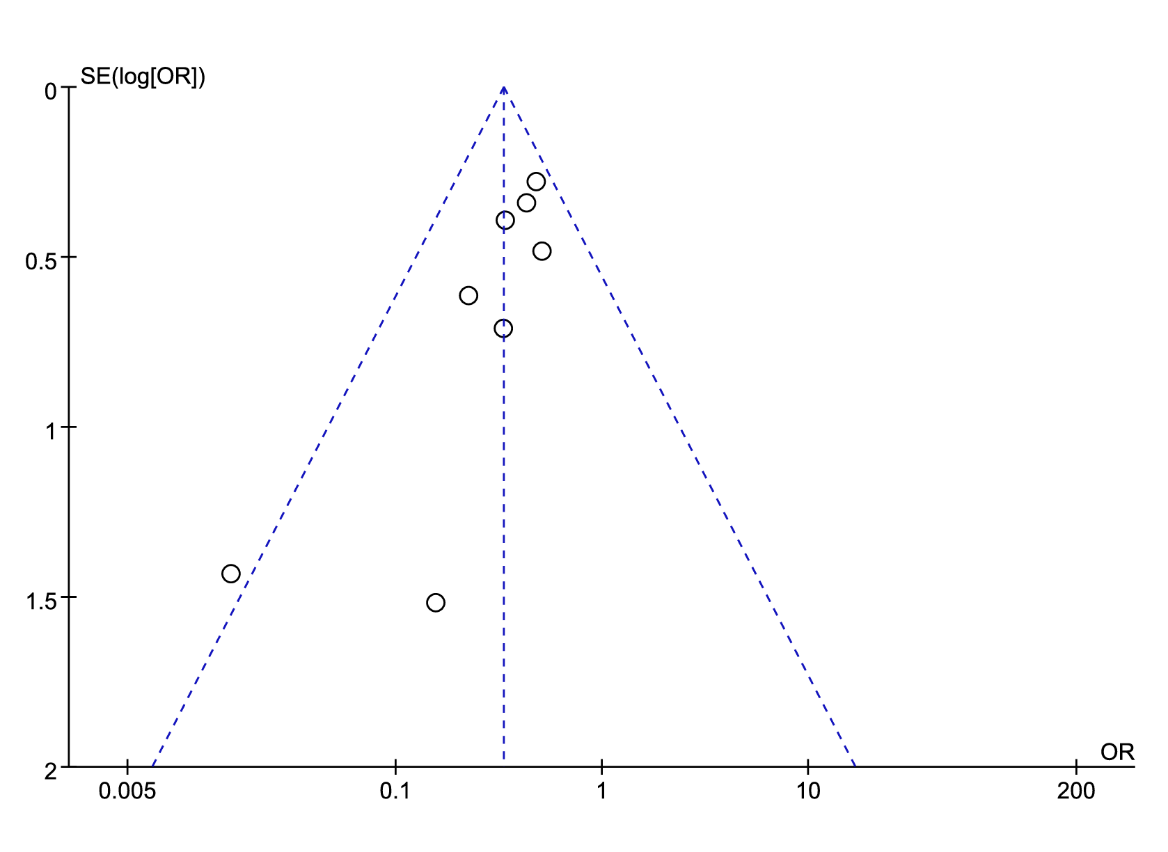


**Additional file 8**

**Table. Summary of adverse events of the low-dose group vs. standard group in the included studies**

| Study |  | A | | B | | C | | D | | E | | F | | G | | H | | I | | J | | K | |
| --- | --- | --- | --- | --- | --- | --- | --- | --- | --- | --- | --- | --- | --- | --- | --- | --- | --- | --- | --- | --- | --- | --- | --- |
|  | LD/SD | LD | SD | LD | SD | LD | SD | LD | SD | LD | SD | LD | SD | LD | SD | LD | SD | LD | SD | LD | SD | LD | SD |
| Harada 2021 [26] | 75/145 | 1 | 1 | 3 | 10 |  |  | 0 | 9 | 0 | 9 |  |  | 2 | 3 | 1 | 5 | 0 | 5 | 0 | 0 | 8 | 35 |
| Yamashita 2021 [35] | 36/45 | 0 | 5 | 0 | 6 | 2 | 6 | 3 | 6 | 3 | 6 |  |  | 1 | 2 |  |  | 0 | 5 | 0 | 0 |  |  |
| Otani 2021 [29] | 74/244 |  |  | 1 | 5 | 0 | 1 | 2 | 3 | 0 | 7 | 0 | 8 | 2 | 9 | 2 | 13 | 0 | 13 | 0 | 0 | 7 | 59 |
| Ohmura 2024 [37] | 60/126 | 0 | 7 | 0 | 18 |  |  |  |  |  |  |  |  |  |  | 0 | 2 | 0 | 9 |  |  | 8 | 46 |
| Utsunomiya 2020 [12] | 105/58 | 0 | 2 | 3 | 5 | 1 | 1 | 1 | 1 | 7 | 9 | 1 | 6 | 1 | 3 | 1 | 3 | 8 | 6 | 0 | 0 | 51 | 34 |
| Shimizu 2019 [31] | 33/65 |  |  |  |  |  |  |  |  |  |  |  |  |  |  |  |  |  |  | 2 | 17 | 6 | 41 |
| Suyama 2016 [32] | 28/31 | 1 | 8 | 3 | 10 |  |  |  |  |  |  |  |  |  |  |  |  | 1 | 6 |  |  | 3 | 13 |
| Yamamoto 2014 [34] | 17/18 |  |  | 0 | 3 |  |  |  |  | 1 | 1 |  |  | 1 | 2 | 0 | 2 | 1 | 1 | 0 | 0 |  |  |
| Takenaka 2012 [33] | 13/28 |  |  | 1 | 2 |  |  | 1 | 1 | 2 | 3 | 2 | 6 |  |  | 4 | 13 | 0 | 1 | 0 | 1 | 4 | 13 |
| Waki 2021 [36] | 167/40 |  |  |  |  |  |  |  |  |  |  |  |  |  |  |  |  |  |  | 2 | 0 |  |  |
| Yamanaga [37] | 51/13 |  |  |  |  |  |  |  |  |  |  |  |  |  |  |  |  |  |  | 0 | 0 | 0 | 0 |
| Chen 2022 [19] | 1193 | 7 |  |  |  | 2 |  | 19 |  |  |  |  |  |  |  |  |  | 17 |  | 0 |  |  |  |
| Shan 2024 [38] | 1763 |  |  |  |  |  |  |  |  |  |  |  |  |  |  |  |  |  |  |  |  |  |  |
| Peterson 2020 [30] | 228 |  |  |  |  |  |  | 1 |  |  |  |  |  |  |  | 3 |  |  |  | 0 |  | 9 |  |
| Zmarlicka 2015 [21] | 77 |  |  |  |  |  |  |  |  |  |  |  |  |  |  |  |  |  |  | 0 |  |  |  |
| Maezawa 2012 [26] | 539 | 20 |  | 12 |  |  |  | 15 |  |  |  | 4 |  |  |  |  |  | 15 |  |  |  |  |  |
| Muto 2011[27] | 156 |  |  |  |  |  |  | 1 |  |  |  |  |  |  |  |  |  | 1 |  | 0 |  | 2 |  |

A = fever; B = rash; C = anemia; D = leukopenia; E = thrombocytopenia; F = hyponatremia; G = hyperkalemia; H = increased serum creatinine; I = liver injury; J = PJP infection; K=total adverse events.

**Additional File 9**

**Funnel plot of adverse events**


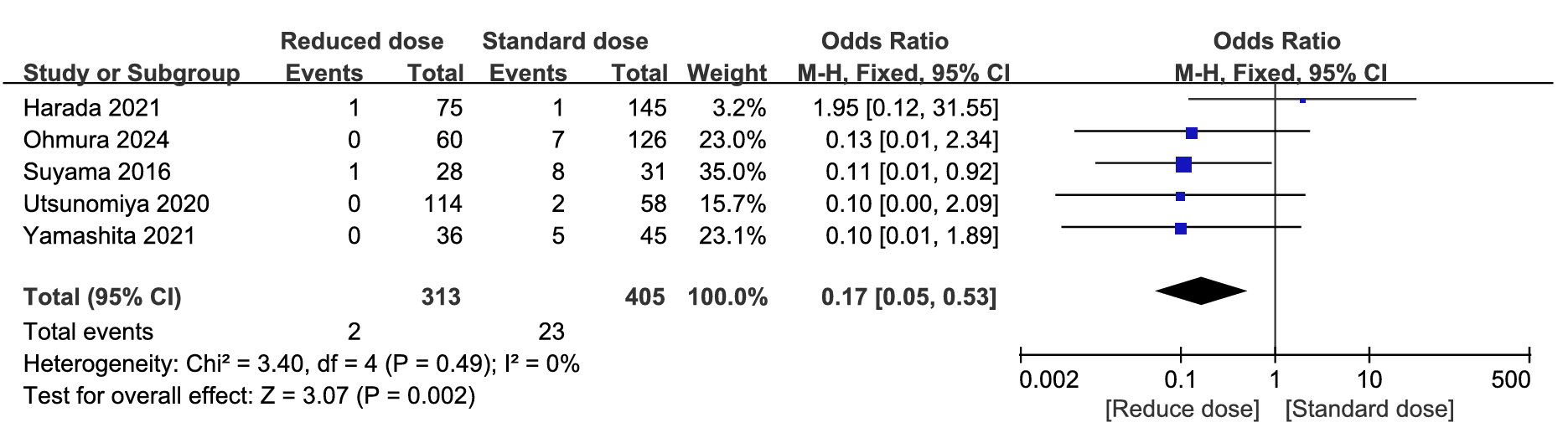


**Figure S1 Forest plots of fever of low-dose vs. standard dose groups.**


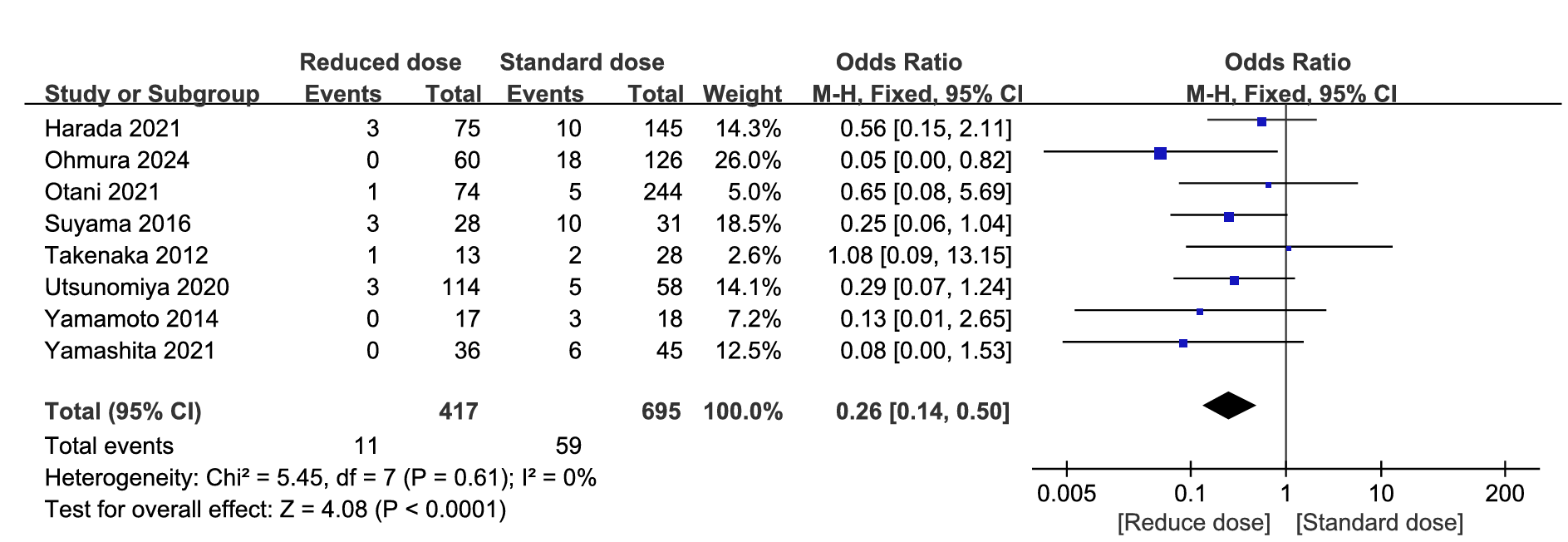


**Figure S2 Forest plots of rash of low-dose vs. standard dose groups.**


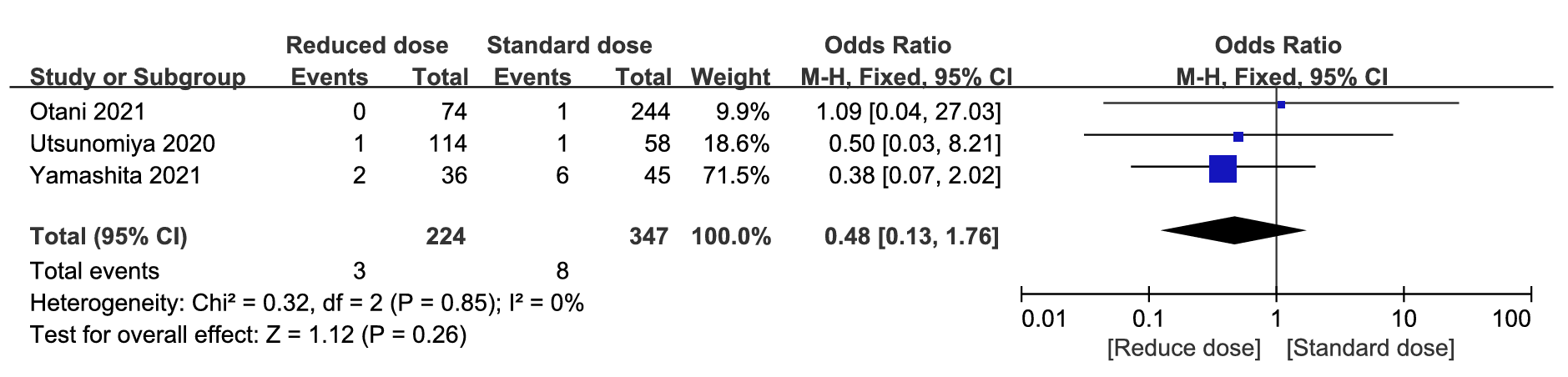


**Figure S3 Forest plots of anemia of low-dose vs. standard dose groups.**


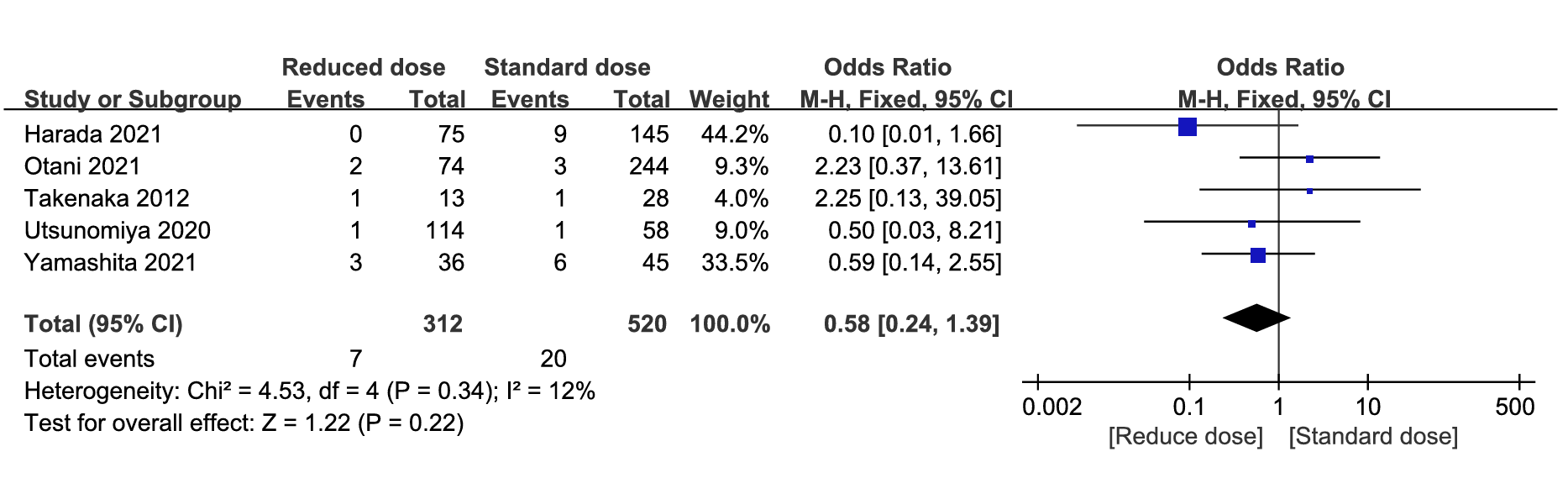


**Figure S4 Forest plots of leuco-cytopenia of low-dose vs. standard dose groups.**


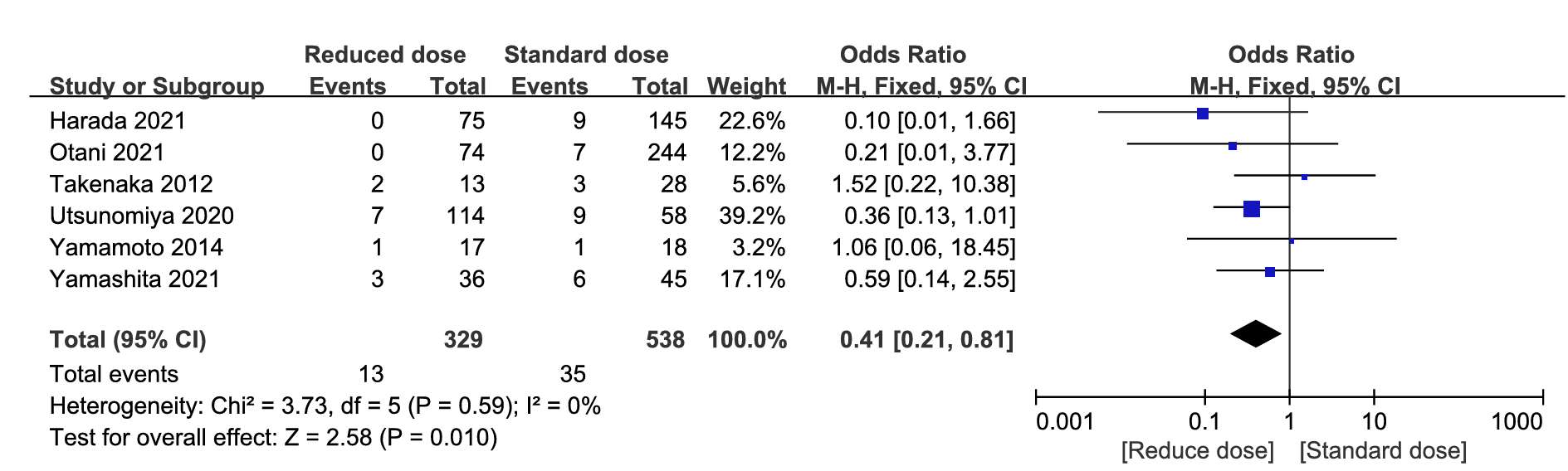


**Figure S5 Forest plots of thrombocytopenia of low-dose vs. standard dose groups.**


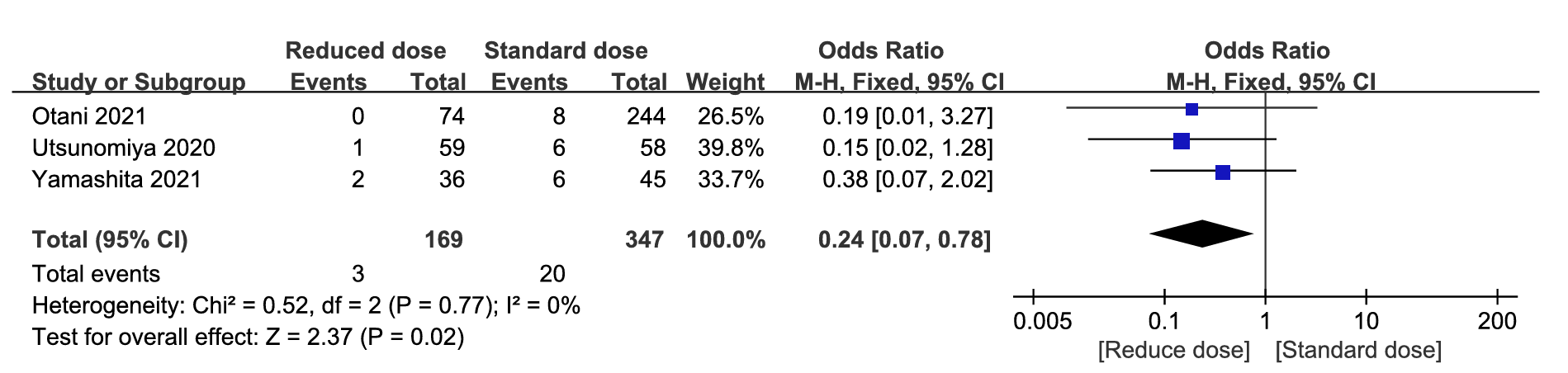


**Figure S6 Forest plots of hyponatremia of low-dose vs. standard dose groups.**


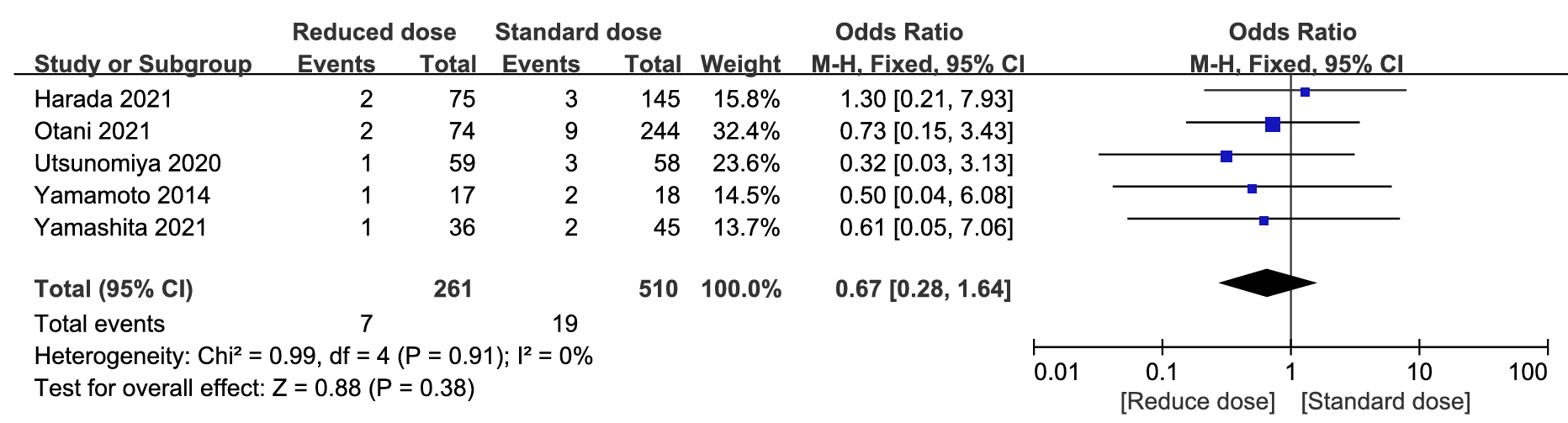


**Figure S7 Forest plots of hyperpotassemia of low-dose vs. standard dose groups.**


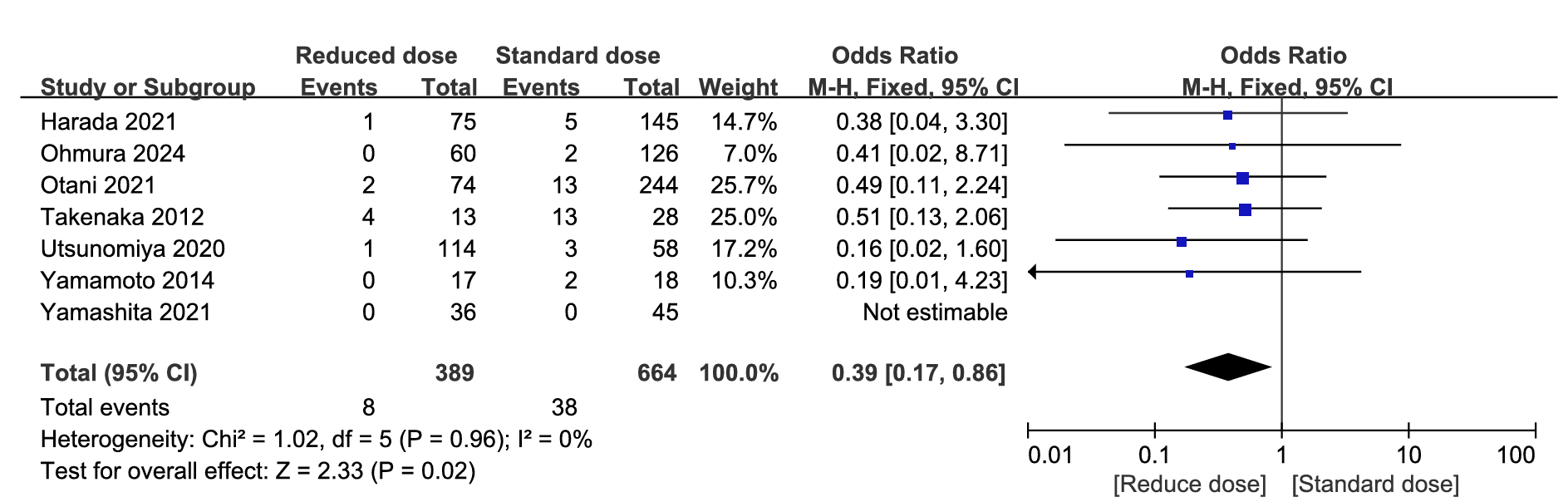


**Figure S8 Forest plots of renal dysfunction of low-dose vs. standard dose groups.**


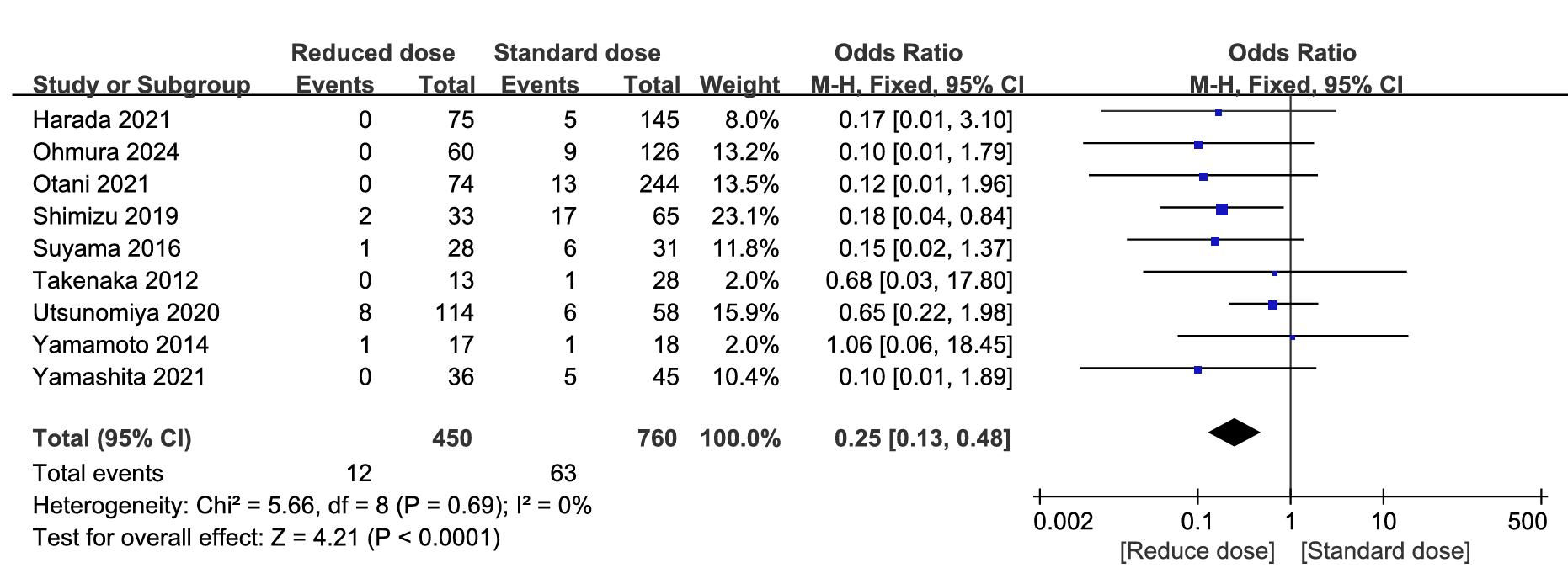


**Figure S9 Forest plots of liver dysfunction of low-dose vs. standard dose groups.**
